# The impact of lookback windows on the prevalence and incidence of chronic diseases among people living with HIV: an exploration in administrative health data in Canada

**DOI:** 10.1186/s12874-021-01448-x

**Published:** 2022-01-06

**Authors:** Ni Gusti Ayu Nanditha, Xinzhe Dong, Taylor McLinden, Paul Sereda, Jacek Kopec, Robert S. Hogg, Julio S. G. Montaner, Viviane D. Lima

**Affiliations:** 1 British Columbia Centre for Excellence in HIV/AIDS, 608-1081 Burrard Street, Vancouver, BC V6Z 1Y6 Canada; 2 Department of Medicine, Faculty of Medicine, University of British Columbia, Vancouver, Canada; 3 Arthritis Research Canada, Richmond, BC Canada; 4 School of Population and Public Health, University of British Columbia, Vancouver, BC Canada; 5 Faculty of Health Sciences, Simon Fraser University, Burnaby, BC Canada

**Keywords:** Administrative health data, Comorbidities, HIV, Incidence, Lookback window, Prevalence, Bias

## Abstract

**Background:**

We described the impact of different lengths of lookback window (LW), a retrospective time period to observe diagnoses in administrative data, on the prevalence and incidence of eight chronic diseases.

**Methods:**

Our study populations included people living with HIV (*N* = 5151) and 1:5 age-sex-matched HIV-negative individuals (*N* = 25,755) in British Columbia, Canada, with complete follow-up between 1996 and 2012. We measured period prevalence and incidence of diseases in 2012 using LWs ranging from 1 to 16 years. Cases were deemed prevalent if identified in 2012 or within a defined LW, and incident if newly identified in 2012 with no previous cases detected within a defined LW. Chronic disease cases were ascertained using published case-finding algorithms applied to population-based provincial administrative health datasets.

**Results:**

Overall, using cases identified by the full 16-year LW as the reference, LWs ≥8 years and ≥ 4 years reduced the proportion of misclassified prevalent and incidence cases of most diseases to < 20%, respectively. The impact of LWs varied across diseases and populations.

**Conclusions:**

This study underscored the importance of carefully choosing LWs and demonstrated data-driven approaches that may inform these choices. To improve comparability of prevalence and incidence estimates across different settings, we recommend transparent reporting of the rationale and limitations of chosen LWs.

**Supplementary Information:**

The online version contains supplementary material available at 10.1186/s12874-021-01448-x.

## Introduction

Large scale administrative health databases depicting claims related to healthcare utilization have become an indispensable data source in contemporary epidemiologic research [1]. These databases represent real-time clinical care practices and provide an inexpensive alternative to primary data collection, such as national health surveys, facilitating longitudinal population-based studies crucial to public health [2]. In measuring the prevalence and incidence of diseases, however, administrative health data are limited in their ability to establish an individual’s instantaneous health status [3]. Instead, to ascertain prevalent or incident cases of diseases in these databases, relevant healthcare encounters must be searched within a specific retrospective period of time or a ‘lookback window’ (LW) [4, 5]. Therefore, researchers who analyze administrative data must be conscientious in ensuring that the chosen length of this LW provides adequate data observability and produces reliable prevalence and incidence estimates.

With people living with HIV (PLWH) living longer than ever before, administrative data are often used to calculate the prevalence and incidence of chronic diseases in this population, alone and in comparison to their HIV-negative counterparts [6–10]. Without a gold standard method for selecting the proper length of these LWs, published studies utilized various lengths, potentially threatening the internal and external validity of the produced prevalence and incidence estimates [3]. Given that prevalence and incidence estimates play substantial roles in informing policies, intervention investments and resource prioritization [11], evidence on the association between LWs, as a critical component of administrative data study design, and the validity of prevalence and incidence estimation is needed.

This study evaluates the impact of varying lengths of LW on the prevalence and incidence estimates of eight common chronic diseases and quantifies potential misclassification bias associated with shorter LWs. The impact of LWs on the annual prevalence and incidence trends of these diseases was also examined. Using data from a population-based cohort in British Columbia (BC), Canada, these analyses were conducted separately for PLWH and HIV-negative individuals, as their distinct healthcare utilization practices are likely to influence choices surrounding LWs. Ultimately, this study sought to propose replicable data-driven approaches to facilitate a more informed study design that would improve the validity of interpretations and comparisons of findings from administrative data studies.

## Methods

### Data sources

Longitudinal de-identified individual-level data were sourced from the Comparative Outcomes And Service Utilization Trends (COAST) study [12]. COAST comprises all diagnosed adult PLWH and a 10% random sample of the general population in BC followed from 1996 to 2013. Two provincial data sources in BC were confidentially linked to form COAST: i) the BC Centre for Excellence in HIV/AIDS Drug Treatment Program (DTP), which provides demographic, antiretroviral treatment (ART)-related and clinical information of all known PLWH [13]; and ii) Population Data BC [14–19], which houses data from various provincial administrative health databases for all BC residents. The COAST study and the aforementioned data linkages have been described elsewhere [12].

### Study design

#### Study populations

In this retrospective cohort study, eligible individuals were ≥ 19 years old and enrolled in COAST for ≥1 day in 2012 (the last full calendar year in COAST study). To ensure an equal amount of historical data, eligible individuals must have been registered with the BC’s publicly funded healthcare system (i.e., Medical Services Plan [MSP]) since 1996. Eligible PLWH were ART-treated and randomly matched at a 1:5 ratio to HIV-negative individuals by birth year and sex at birth.

#### Chronic diseases

This study focused on eight chronic diseases highly prevalent among PLWH and BC’s general population [10, 20]: cardiovascular diseases (CVD), kidney disease, liver disease, chronic obstructive pulmonary disease (COPD), diabetes, osteoarthritis, hypertension, and Alzheimer’s and/or non-HIV-related dementia (Alzheimer’s/dementia). In both study populations, diagnoses of these diseases were ascertained by the same published case-finding algorithms using International Classification of Disease Ninth (ICD-9) and Tenth Revisions, Canada (ICD-10-CA) diagnosis codes and Canada-wide Drug Identification Numbers. We applied these algorithms to the following BC’s administrative health databases: i) the Discharge Abstract Database [15], which captures hospital discharges and day-surgeries from acute-care hospitals; ii) MSP billings database [16], which captures medically necessary outpatient services provided by physicians, laboratory tests and diagnostic procedures; iii) PharmaNet database [19], which captures prescription drugs dispensed by community and outpatient pharmacies. Supplemental Table 1 details the selected chronic diseases and case-finding algorithms. Following age-restrictions in the case-finding algorithms, individuals considered for hypertension, COPD and Alzheimer’s/dementia analyses were older than 20, 35 and 40 years, respectively.

### Analytical approaches

Analyses were performed using R© software version 3.2.2 (R Core Team, Vienna, Austria) or SAS software version 9.4 (SAS, Cary, North Carolina, United States).

#### Prevalence and incidence estimates

**Fig. 1:**
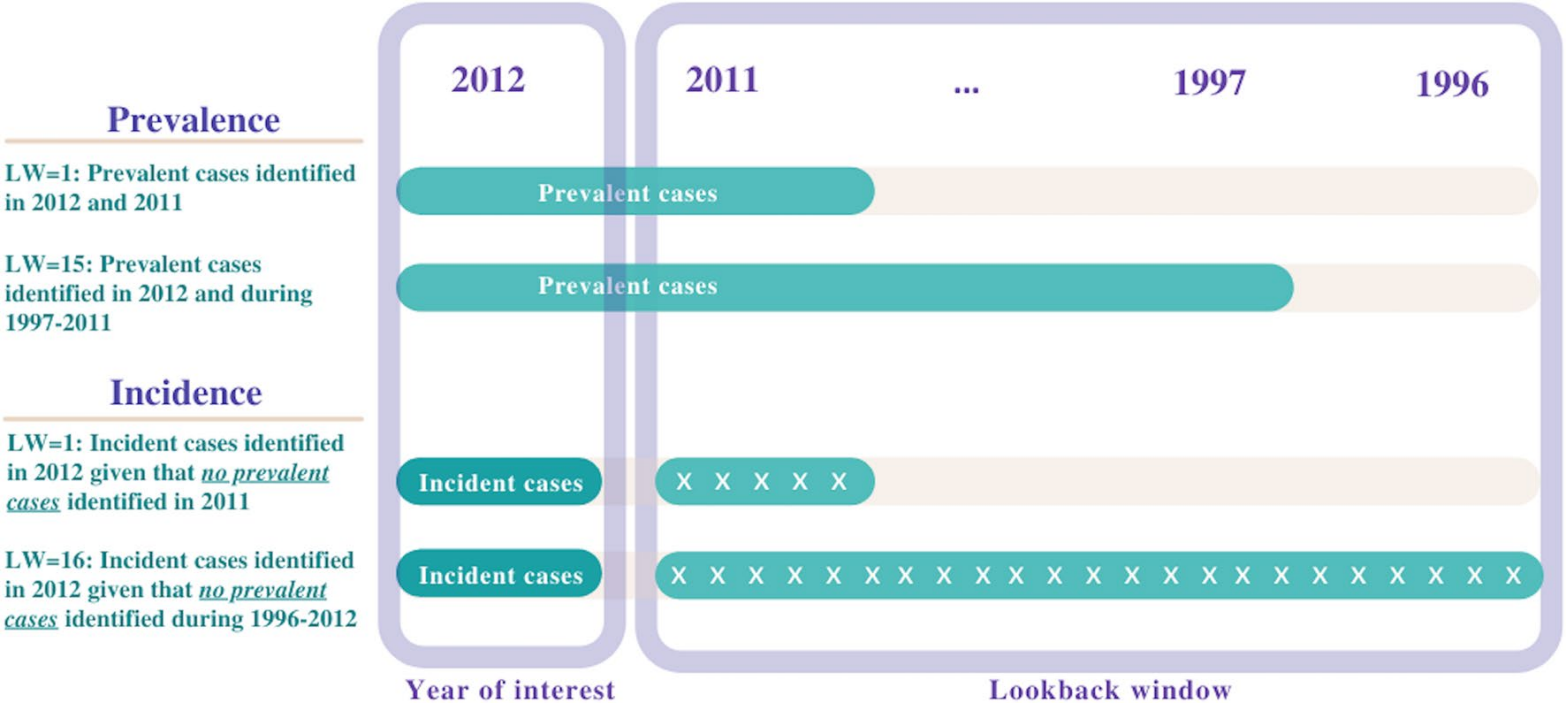
Schematic illustrating the measurement of prevalent and incident cases of chronic diseases for year 2012 using varying lookback windows. Note: LW: lookback window

We estimated period prevalence (hereinafter referred to as prevalence) and cumulative incidence or incidence proportion [21] (hereinafter referred to as incidence) of each chronic disease for the year 2012 using varying LWs. Based on the availability of retrospective data prior to 2012, lengths of the LWs ranged from zero years (i.e., no historical data considered – 2012 only) to 16 years (i.e., historical data from 1996 to 2011 considered as well). Prevalent cases were existing diagnoses identified at any point in 2012 and within a defined LW (Fig. 1). For each LW, prevalence of a particular disease was then calculated as the proportion of individuals present for ≥1 day in 2012 with a prevalent case as defined above. Conversely, incident cases were new diagnoses identified at any point in 2012 provided that no prevalent cases were identified within a defined LW (Fig. 1). Individuals with prevalent cases within a defined LW were thus excluded from an incidence calculation as they were no longer ‘at-risk’ of a new diagnosis. For each LW, incidence was then calculated as the proportion of ‘at-risk’ individuals present for ≥1 day in 2012 with an incident case.

#### Proportion of misclassified prevalent and incident cases

To quantify the bias associated with using shorter LWs, we measured proportion of misclassified prevalent and incident cases of each chronic disease for the year 2012 relative to the reference standard. In the absence of a gold standard or alternative data sources with longer follow-up time, prevalent and incident cases measured using complete retrospective data available in COAST (i.e., a 16-year LW) were considered the reference standard. For prevalence, the proportion of prevalent cases not captured by a shorter LW was calculated, reflecting the extent to which shorter LWs may be associated with an underestimated number of prevalent cases. For incidence, we calculated the proportion of incident cases as captured by a shorter LW that, in fact, were prevalent. This proportion thus demonstrated the extent to which shorter LWs may be associated with an overestimated number of incident cases. As sensitivity analyses, we estimated proportion of misclassified prevalent and incident cases using cases identified using a 10-year and, separately, a 13-year LW as the reference standard.

#### Sub-analysis: annual prevalence and incidence trends

Using varying LWs, we assessed annual prevalence and incidence trends of each chronic disease from 2001 to 2012 in both study populations. This sub-analysis served to examine whether the impact of varying LWs on prevalence and incidence estimates was observed across different years, and whether varying LWs influenced the stability of annual prevalence and incidence trends. Of note, since the earliest retrospective data available was from 1996, the maximum length of LWs varied for each year. For instance, the year 2001 had a maximum of five years of LW, while year 2010 could have up to 14 years.

## Results

Included in our study were 5151 PLWH and 25,755 HIV-negative individuals. The two study populations were matched on age (median in 2012: 50 years, [25th-75th percentile: 44-56]) and sex at birth (82% male). Supplemental Fig. 1 illustrates the stepwise selection process of our final study populations.

### Prevalence and incidence estimates

**Fig. 2:**
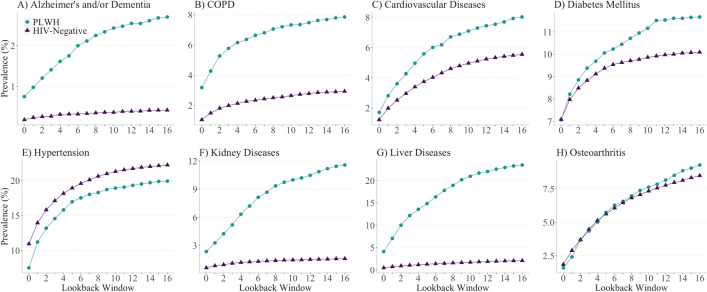
Prevalence chronic diseases among people living with HIV and HIV-negative individuals in British Columbia, Canada for year 2012 across varying lookback windows. Note: PLWH: people living with HIV; COPD: Chronic Obstructive Pulmonary Diseases. Vertical scales differ for each graph for illustration purposes

Consistent in both study populations, prevalence estimates of all chronic diseases for the year 2012 substantially increased as the length of LW increased from zero to 16 years (Fig. 2; data in Supplemental Table 2). Except for osteoarthritis and diabetes, prevalence estimates of all diseases appeared to stabilize when using LWs ≥8 years. For instance, the estimated prevalence of hypertension among PLWH using one-, eight- and 16-year LWs were 11.2, 18.3 and 19.9%, respectively, suggesting a minor influence of LWs on the prevalence estimates when LW expanded from eight to 16 years. For all diseases, except hypertension and osteoarthritis, the difference in prevalence estimates between PLWH and their HIV-negative counterparts increased as the length of LW increased. Prevalence estimates of kidney diseases in PLWH, for instance, were 2.5% higher than HIV-negative individuals when using one-year LW, but became 10.0% higher when using 16-year LW. Similarly, for liver diseases, the gap in the prevalence estimates grew from 6.4 to 21.4% when using one-year versus 16-year LW, respectively.

**Fig. 3:**
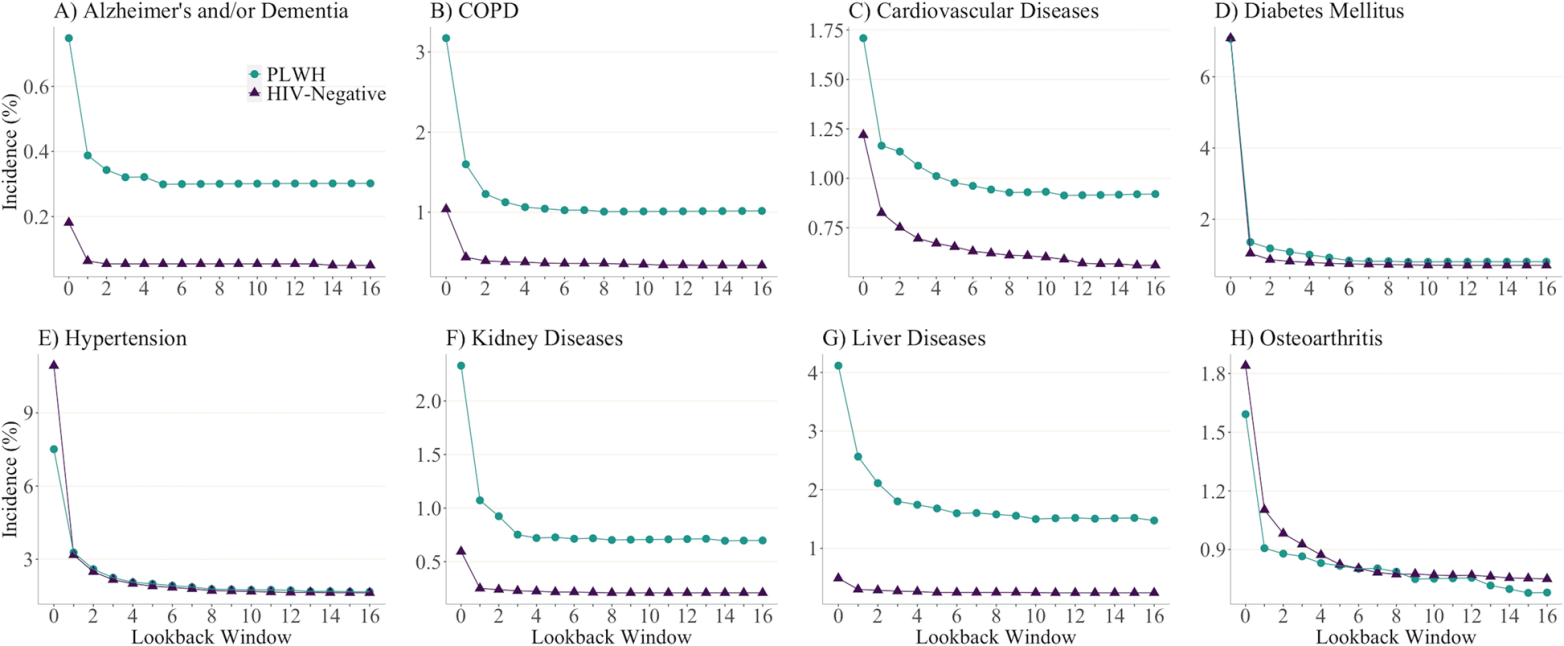
Incidence of chronic diseases among people living with HIV and HIV-negative individuals in British Columbia, Canada for year 2012 across varying lookback windows. Note: PLWH: people living with HIV; COPD: Chronic Obstructive Pulmonary Diseases. Vertical scales differ for each graph for illustration purposes

Contrary to prevalence estimates, incidence estimates of all chronic diseases for the year 2012 substantially decreased as the length of LW increased from zero to 16 years (Fig. 3; data in Supplemental Table 3). Moreover, incidence estimates of most diseases appeared to stabilize when LWs ≥4 years were applied. For instance, the estimated incidence of COPD among PLWH using one-, four- and 16-year LWs were 1.6, 1.1 and 1.0%, respectively, suggesting a minor influence of LWs on the incidence estimates when LW expanded from four or 16 years. Longer LWs, however, may be warranted for more stable incidence estimates of cardiovascular diseases and osteoarthritis. Unlike prevalence estimates, the difference in incidence estimates between PLWH and their HIV-negative counterparts remained consistent across varying LWs.

### Proportion of misclassified prevalent and incident cases

As outlined in Table 1, in 2012, proportion of misclassified prevalent cases by shorter LWs (relative to the 16-year LW reference standard) varied substantially across diseases. For instance, in both study populations, using LWs ≥3 years resulted in ≥80% of prevalent cases (i.e., < 20% misclassification) of diabetes being captured. In contrast, for liver diseases and osteoarthritis, LWs ≥9 years were required to achieve comparable results. Differences in the proportion of misclassified prevalent cases between PLWH and HIV-negative individuals generally narrowed with longer LWs. Except for Alzheimer’s/dementia and kidney diseases, the absolute difference in proportion of misclassified prevalent cases between PLWH and HIV-negative individuals across all LWs was, in most cases, < 5%. Consistent with the 16-year LW reference standard, when a 10-year (Supplemental Table 4) or a 13-year reference standard (Supplemental Table 5) was employed, LWs ≥8 years reduced the proportion of misclassified prevalent cases of most diseases to < 20%.

**Table 1:**
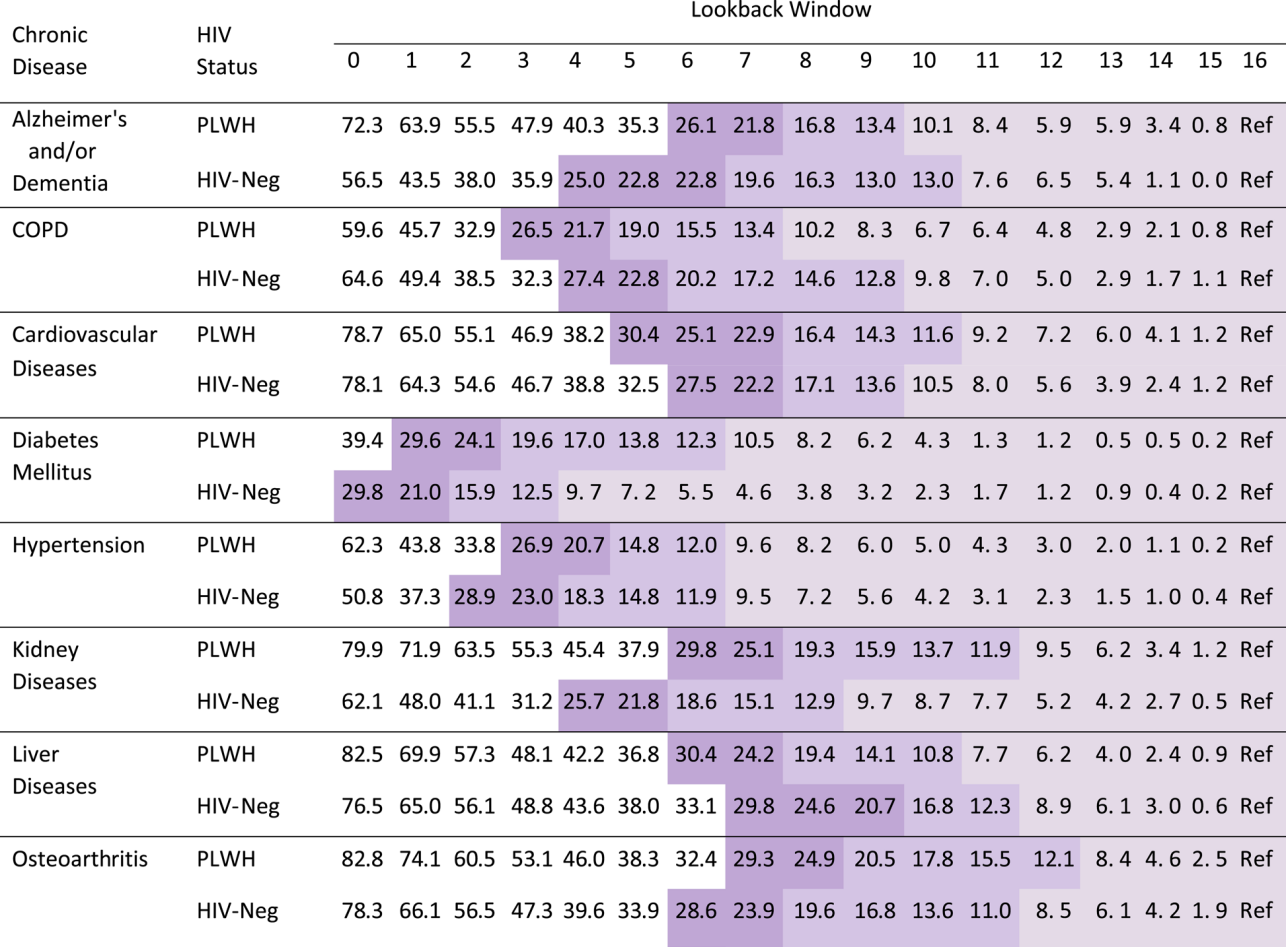
Proportion of misclassified prevalent cases (as percentages, relative to the 16-year lookback window) of chronic diseases among people living with HIV and HIV-negative individuals in 2012 across varying lookback windows

Note: PLWH: people living with HIV; HIV-Neg: HIV-negative individuals; COPD: Chronic Obstructive Pulmonary Diseases; Ref: reference. Lightest, medium and darkest shade represent proportion of misclassification < 10, < 20 and < 30%, respectively

Proportion of misclassified prevalent cases was proportion of prevalent cases (i.e., identified using 16-year lookback window) not captured by a shorter lookback window

Proportion of misclassified incident cases also varied substantially across diseases (Table 2). In both study populations, extending the length of LW from four to six years reduced the proportion of misclassified incident cases for most diseases from < 20 to < 10%. Exceptions included liver diseases and osteoarthritis among PLWH where LWs ≥9 and ≥ 13 years were needed, respectively, to reduce the proportion of misclassified incident cases to < 10%. Across all LWs, the absolute difference in proportion of misclassified incident cases between PLWH and HIV-negative individuals was largely < 5% for all diseases except liver diseases. Note that LWs ≥4 years also reduced the proportion of misclassified incident cases of most diseases to < 20% relative to the 10-year (Supplemental Table 6) or the 13-year LW (Supplemental Table 7) reference standard.

**Table 2:**
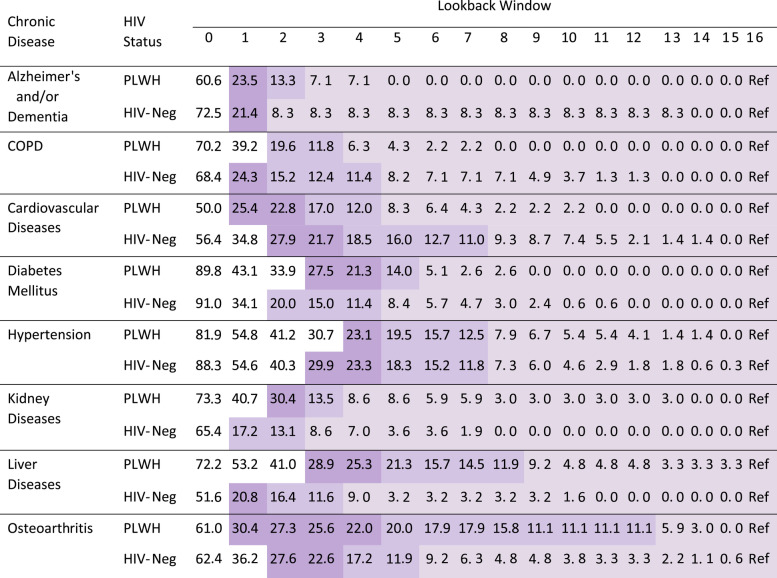
Proportion of misclassified incident cases (as percentages, relative to the 16-year lookback window) of chronic diseases among people living with HIV and HIV-negative individuals in 2012 across varying lookback windows

Note: PLWH: people living with HIV; HIV-Neg: HIV-negative individuals; COPD: Chronic Obstructive Pulmonary Diseases; Ref: reference. Lightest, medium and darkest shade represent proportion of misclassification < 10, < 20 and < 30%, respectively

Proportion of misclassified incident cases was proportion of incident cases as captured by a shorter lookback window that, in fact, were prevalent (i.e., when identified using 16-year lookback window)

### Sub-analysis: annual prevalence and incidence trends

**Fig. 4:**
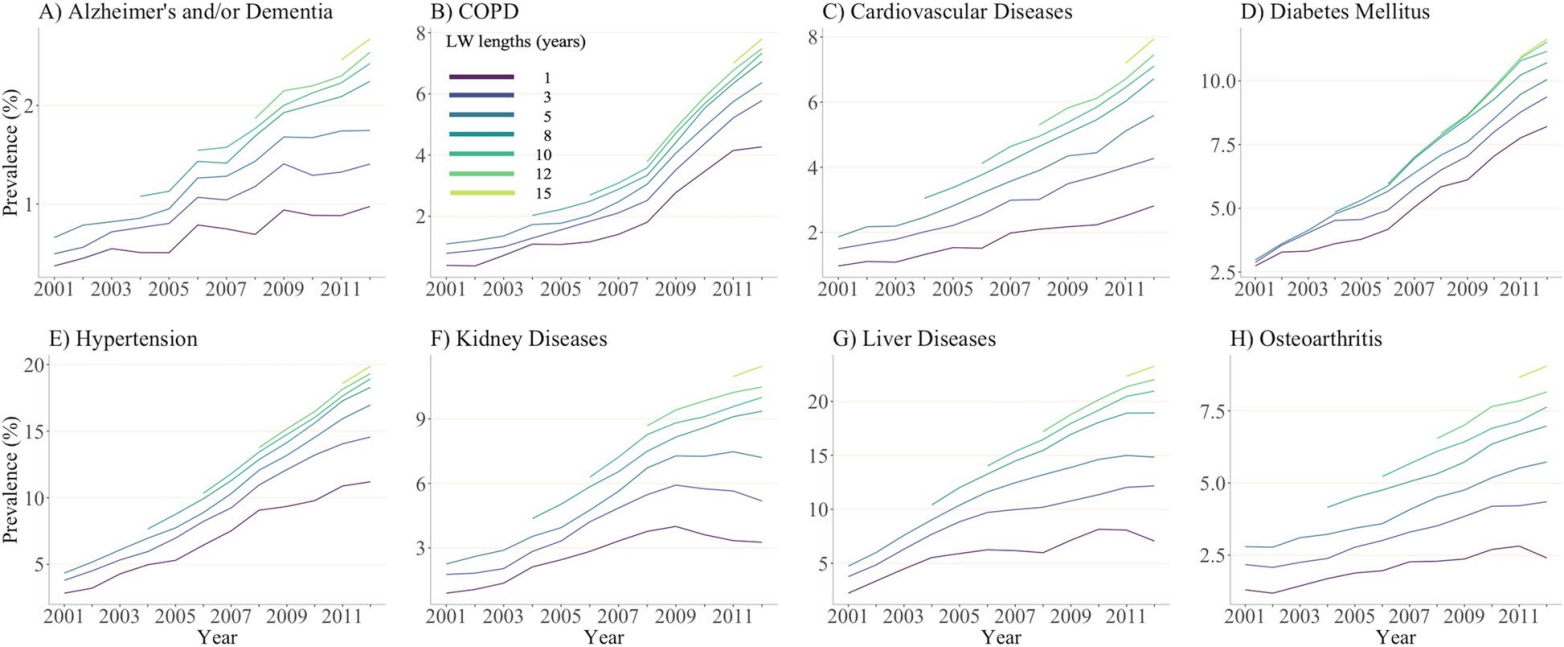
Annual trends in prevalence of chronic diseases among people living with HIV in British Columbia from 2001 to 2012 using varying lookback windows. Note: PLWH: people living with HIV; LW: lookback window; COPD: Chronic Obstructive Pulmonary Diseases. Vertical scales differ for each graph for illustration purposes. Supplemental Fig. 2 shows similar illustration for HIV-negative individuals

**Fig. 5:**
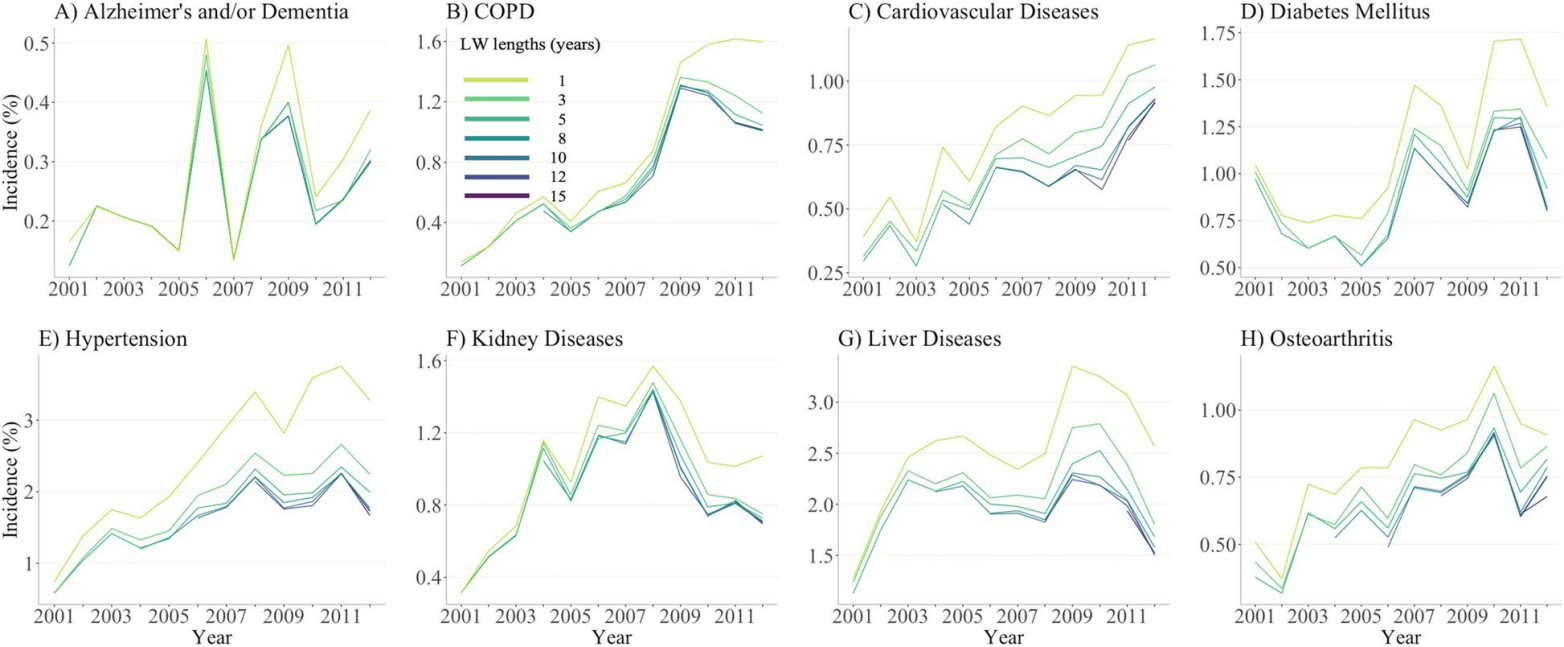
Annual trends in incidence of chronic diseases among people living with HIV in British Columbia from 2001 to 2012 using varying lookback windows. Note: PLWH: people living with HIV; COPD: Chronic Obstructive Pulmonary Diseases. Vertical scales differ for each graph for illustration purposes. Supplemental Fig. 3 shows similar illustration for HIV-negative individuals

Similar to the 2012 estimates, with longer LWs, prevalence estimates of chronic diseases for each year between 2001 and 2011 consistently increased in both populations (Supplemental Table 8 and 9), while incidence estimates decreased (Supplemental Table 10 and 11). The impact of LWs on annual prevalence and incidence trends, however, indicated some population-specific and disease-specific patterns with shorter LWs leading to unstable trends. For example, among PLWH, prevalence trends of kidney diseases between 2009 and 2012 appeared to decrease when using LWs < 3 years, flatten when using a five-year LW and increase when using LWs ≥9 years (Fig. 4). Similarly, incidence trends of COPD among PLWH between 2009 and 2012 appeared to increase when using a one-year LW, but decrease when using LWs ≥4 years (Fig. 5). Supplemental Fig. 2 and 3 illustrate prevalence and incidence trends among HIV-negative individuals.

## Discussion

Using a large provincial health claim database which spanned over 17 years, this study highlights the impact of lengths of LWs on prevalence and incidence estimates of eight chronic diseases among PLWH and a sample of age-sex-matched HIV-negative individuals. Compared to 16-year LW, shorter LWs may fail to capture some prevalent cases, leading to underestimated prevalence estimates. In contrast, shorter LWs may lead to overestimated incidence estimates since some prevalent cases may be misclassified as incident. The impact of LWs on prevalence and incidence estimation was disease- and population-specific. Given the potential implications of prevalence and incidence measures in health policies and program developments, this study underscored the importance of LW choices and presented a methodological approach that may inform these choices. Furthermore, this study recommends the practice of reporting the rationale behind LW choices as a key component of study design in administrative data research, promoting more robust and cautious comparisons with prevalence and incidence estimates across various studies.

With longer LWs, prevalence and incidence estimates of most diseases stabilized, and, simultaneously, the proportion of misclassified prevalent and incident cases became marginal. In our study populations, the minimum length of LW to capture ≥80% of prevalent cases (i.e., cases identified by the 16-year LW) varied from three years for diabetes to nine years for osteoarthritis and liver diseases. These differences may be explained by healthcare-seeking practices related to the treatment and management of these diseases, as well as the robustness of the applied case-finding algorithms in capturing these practices within a defined LW. For instance, in addition to hospitalizations and physician visits, diagnoses of diabetes were also identifiable through prescriptions of insulin, metformin and other oral antihyperglycemic, allowing the detection of current diabetes cases within a shorter observation period. In contrast, both osteoarthritis and liver diseases were only identified from hospitalizations and physician visits, and the longer LWs required to capture ≥80% of prevalent cases (as defined by the maximum 16-year LW) may insinuate that the management of these diseases did not necessitate frequent healthcare encounters or that patients did not seek care as frequently. Osteoarthritis, for instance, is commonly managed through over-the-counter pain-relieving medications and rehabilitation therapies (e.g., physical and occupational therapies) [22], which are not captured in the available provincial claim databases. Meanwhile, the prevalence of decompensated and compensated cirrhosis as well as chronic liver diseases among BC’s PLWH has been found significantly higher among those with history of injection drug use [10], a population who historically face additional barriers to access health services [23, 24].

For most diseases, a minimum LW of four and six years were required to reduce the proportion of misclassified incident cases to < 20 and < 10%, respectively, with longer LWs needed for liver diseases and osteoarthritis. This observation is aligned to that of prevalence, whereby, the postulated infrequent healthcare encounters associated with liver diseases and osteoarthritis called for a longer disease-free period to ascertain that prevalent cases are not misclassified as incident. Note that the length of LW affects both the numerator of incidence estimates (i.e., the number of incident cases), and the denominator (i.e., the number of ‘at-risk’ individuals, which were those without previously identified prevalent cases). As such, the impact of LW choices on overall incidence estimates was lessened, with incidence estimates of most diseases stabilized when LWs ≥4 years were utilized.

Comparing PLWH and HIV-negative individuals, gaps in prevalence estimates of most diseases increased as the length of LW increased, while gaps in incidence estimates remained constant. The observed disparities in the impact of LWs on prevalence estimates were likely driven by PLWH’s higher engagement with the healthcare system and earlier diagnosis of some chronic conditions [10], thus receiving these diagnoses earlier in the study period. Despite the gaps in prevalence and incidence estimates, for most diseases, the observed gaps in the proportion of misclassified prevalence cases and incidence cases between PLWH and HIV-negative individuals were < 5%, which may justify the use of identical LWs when comparing the two populations. The observed inconsistent prevalence and incidence trends among PLWH, particularly when applying LWs < 5 years, further confirmed the population-specific impact of varying LW lengths and reiterated the importance of careful consideration of LWs.

Our findings complemented the current literature in that prevalence estimates of five chronic conditions, including COPD, among commercially insured and Medicare patients in the United States were underestimated when a one-year as opposed to a two-year LW was employed [3]. Furthermore, claims data studies have also noted that shorter LWs likely overestimated incidence estimates of various chronic conditions [3, 25–30]. Over a nine-year study period, a German study demonstrated that LWs ≥4 years were necessary to allow < 10% overestimation of incidence of diabetes [26]. While looking at hospitalizations over a 12-year period, an Australian study signified that a one-year LW was sufficient to reduce misclassified incident stroke cases to < 10% [27]. Lastly, a three-year Medicare-based study found that the impact of shortening LWs on incidence rates of chronic diseases, including COPD, diabetes and hypertension, was stronger among women with breast cancer compared to those without [29]. This finding corroborated ours in that the impact of LW choices on disease estimates may be influenced by a population’s healthcare engagement level.

With over 17 years of data, our study further elucidates the impact of LW choices on prevalence and incidence estimates of chronic conditions. To mitigate administrative data’s susceptibility to coding errors, we employed case-finding algorithms incorporating relevant hospitalizations, physician visits and drug prescriptions to ascertain chronic disease diagnoses. Whenever possible, we used published algorithms by BC Ministry of Health, which were consistent with the Canadian Chronic Disease Surveillance System case definitions and further informed by epidemiology and medical care experts considering BC-specific claims-related practices [20, 31]. Note that this study did not intend to measure the performance of these case-finding algorithms. Instead, within BC’s universal healthcare context, we examined the impact of LW choices in both PLWH and HIV-negative individuals. As PLWH age, studies comparing the burden of chronic diseases in PLWH and HIV-negative individuals are common, and our findings highlight important methodological considerations. For instance, as opposed to utilizing identical LWs, employing different LWs with similar proportion of misclassified prevalent or incident cases for each population may lead to minimal differential misclassification bias by HIV status.

The data-driven approaches presented in this study are straightforward and replicable to other settings, and can serve as exploratory analyses which guide administrative data researchers as they choose appropriate LWs for the diseases and populations of interest. The choice of these LWs is crucial as inappropriate LWs may pose threat on the internal and external validity of the produced prevalence and incidence estimates [3], with potentially disadvantageous downstream consequences. For instance, in examining the effect of comorbidities on patients’ health outcomes and healthcare utilization, suboptimal LWs may underestimate the number of prevalent cases, thus weakening the study’s statistical power and jeopardizing the accuracy and precision of the measured effect sizes. Similarly, suboptimal LWs may misclassify prevalent cases as incident. This misclassification, for instance, may lead to unreliable measurement of effect sizes, such as the risk of developing a particular disease, where the distinction between incident and prevalent cases is critical. With ineffective health policies, off-target intervention prioritization and inadequate resource allocation being among the wider consequences of improper estimation of prevalence and incidence, specific attention to LW choices as a key component of study design in administrative data research is thus warranted.

Note that, with no gold standard or alternative data sources, we employed our study’s longest available LW (i.e., 16 years) as the reference standard, which limited the interpretations of our findings. Readers should keep in mind that, unlike data such as participants’ health history from birth, the prevalence and incidence estimated using the 16-year LW may not be completely unbiased. As a result, the proportion of misclassified cases calculated against the 16-year LW’s estimates may not directly quantify the true misclassification bias or the validity of measures associated with shorter LWs. Nonetheless, as most of the observed prevalence and incidence stabilized when LWs ≥8 years were used, the prevalence and incidence reference standards estimated using the 16-year LW remained an apt proxy of the true measures, and our findings remained valuable in elucidating the impact of LWs on the consistency of prevalence and incidence estimates.

Additional limitations should be considered. First, our study population was limited to participants with a complete follow-up within our population-based cohort. In practice, the choice of LWs is likely a compromise between minimizing misclassification bias and maximizing sample size (i.e., maximizing the number of participants possessing sufficient LW data) [30, 32]. In removing the influence of fluctuating sample size, our study design allowed for assessment of misclassification bias in prevalence and incidence estimates associated with varying LW lengths only. Second, our study focused on chronic diseases and, thus, our approaches would not be applicable in the context of infectious diseases. Third, to maximize sample size, several conditions were analyzed together within major disease groups such as CVD and liver diseases. The impact of varying LWs observed within these disease groups, therefore, was likely an averaged impact that should not be directly interpreted for each individual condition in these disease group. Fourth, a general limitation to administrative data included the inability to detect asymptomatic and undiagnosed cases, which would affect the calculated proportion of misclassified cases. BC’s universal healthcare context, however, minimized barriers to access healthcare and, in turn, improved likelihood of diagnoses. Consequently, caution is warranted when interpreting our findings in non-universal healthcare settings. Fifth, the last full calendar year of data available to this study was for 2012. While newer data may reflect more contemporary healthcare seeking practices, our study nonetheless serves as a replicable exercise emphasizing the importance of carefully choosing LWs. Lastly, factors such as sex, gender identity, age and ethnicity also affect healthcare seeking practices [33]. Therefore, the impact of LWs on prevalence and incidence estimates may vary across these population subgroups. Although we did not stratify our analyses based on these factors, our study comparing PLWH and HIV-negative individuals elucidate how differential healthcare seeking behaviors may influence the impact of LWs on prevalence and incidence estimates.

## Conclusions

In summary, this study underlined the consequences of varying lengths of LW on the internal validity of prevalence and incidence estimates of eight common chronic diseases among PLWH and HIV-negative individuals. We demonstrated reproduceable data-driven approaches that quantified misclassification bias associated with shorter LWs to assist researchers in deciding an optimal study design, with additional considerations for studies comparing different populations. Given the potential ramifications of inaccurate prevalence and incidence measures on health policies and program developments, we support the transparent reporting of LW choices as a key component of administrative data study design, along with the rationale and limitations to these choices. A thoughtful and clearly reported LW strengthens a study’s internal validity by promoting uniformity in observations across study participants, hence removing potential bias arising from differential data availability, and enabling rigorous interpretation of prevalence and incidence estimates. External validity would also be strengthened, allowing for careful comparisons with estimates from other settings. To further elucidate proper LWs for prevalence and incidence estimates, future studies should delve into healthcare utilization practices associated with diagnoses and long-term management of specific chronic diseases, as well as consider changes in any population-specific clinical guidelines or healthcare policies over the years that may affect the likelihood of diagnosis and patterns of relevant healthcare encounters.

## Supplementary Information

**Additional file 1.**

## Data Availability

The British Columbia Centre for Excellence in HIV/AIDS (BC-CfE) is prohibited from making this data set available publicly due to prohibitions in the information sharing agreement under which the data stewards provided the data to the BC-CfE. The underlying analytical codes are available from the authors on request.

## Abbreviations

ART: Antiretroviral treatment
BC: British Columbia
COAST: Comparative Outcomes And Service Utilization Trends study
COPD: Chronic obstructive pulmonary disease
CVD: Cardiovascular diseases
DTP: BC Centre for Excellence in HIV/AIDS Drug Treatment Program
ICD-9: International Classification of Disease Ninth Revision
ICD-10-CA: International Classification of Disease Tenth Revision, Canada
LW: Lookback window
PLWH: People living with HIV

## References

[1] Gavrielov-Yusim N, Friger M. Use of administrative medical databases in population-based research. J Epidemiol Community Heal. 2014; [cited 2021 May 16]; Available from: http://jech.bmj.com/. 10.1136/jech-2013-202744 24248997

[2] Schneeweiss S, Avorn J (2005). A review of uses of health care utilization databases for epidemiologic research on therapeutics. J Clin Epidemiol.

[3] Rassen JA, Bartels DB, Schneeweiss S, Patrick AR, Murk W (2019). Measuring prevalence and incidence of chronic conditions in claims and electronic health record databases. Clin Epidemiol.

[4] Schneeweiss S (2007). Understanding secondary databases: a commentary on “Sources of bias for health state characteristics in secondary databases”. Journal of Clinical Epidemiology.

[5] Nakasian SS, Rassen JA, Franklin JM (2017). Effects of expanding the look-back period to all available data in the assessment of covariates. Pharmacoepidemiol Drug Saf.

[6] Rasmussen LD, May MT, Kronborg G, Larsen CS, Pedersen C, Gerstoft J (2015). Time trends for risk of severe age-related diseases in individuals with and without HIV infection in Denmark: A nationwide population-based cohort study. Lancet HIV.

[7] Kong AM, Pozen A, Anastos K, Kelvin EA, Nash D (2019). Non-HIV comorbid conditions and polypharmacy among people living with HIV age 65 or older compared with HIV-negative individuals age 65 or older in the United States: A retrospective claims-based analysis. AIDS Patient Care STDS.

[8] Christensen S, Wolf E, Altevers J, Diaz-Cuervo H (2019). Comorbidities and costs in HIV patients: A retrospective claims database analysis in Germany. PLoS One.

[9] Yang HY, Beymer MR, Chuan SS (2019). Chronic Disease Onset Among People Living with HIV and AIDS in a Large Private Insurance Claims Dataset. Sci Rep.

[10] Nanditha NGA, Paiero A, Tafessu HM, St-Jean M, McLinden T, Justice AC, et al. Excess burden of age-associated comorbidities among people living with HIV in British Columbia, Canada: A population-based cohort study. BMJ Open. 2021;11(1):e041734. 10.1136/bmjopen-2020-041734. Available from: https://pubmed.ncbi.nlm.nih.gov/33419911/. [cited 2021 Mar 21]. 10.1136/bmjopen-2020-041734 PMC7799128 33419911

[11] Nsubuga P, White ME, Thacker SB, Anderson MA, Blount SB, Broome CV, Jamison DT, Breman JG, Measham AR, Alleyne G, Claeson M, Evans DB (2006). Public Health Surveillance: A Tool for Targeting and Monitoring Interventions “What gets measured gets done.”-Anonymous. Disease Control Priorities in Developing Countries [Internet].

[12] Eyawo O, Hull MW, Salters K, Samji H, Cescon A, Sereda P (2018). Cohort profile: the Comparative Outcomes And Service Utilization Trends (COAST) Study among people living with and without HIV in British Columbia, Canada. BMJ Open.

[13] British Columbia Centre for Excellence in HIV/AIDS (BCCfE). Drug Treatment Program [Internet]. 2019 [cited 2018 Apr 24]. Available from: http://www.cfenet.ubc.ca/drug-treatment-program%0A; http://www.cfenet.ubc.ca/research/laboratory-program.

[14] British Columbia Ministry of Health [creator] (2014). Consolidation File (MSP Registration & Premium Billing). V2. Population Data BC [publisher]. Data Extract. MOH (2014). [cited 2021 Mar 2].

[15] Canadian Institute for Health Information [creator] (2014). Discharge Abstract Database (Hospital Separations). V2. Population Data BC [pub- lisher]. Data Extract. MOH (2014). [cited 2021 Mar 2].

[16] British Columbia Ministry of Health [creator] (2014). Medical Services Plan (MSP) Payment Information File. V2. Population Data BC [publisher]. Data Extract. MOH (2014). [cited 2021 Mar 2].

[17] British Columbia Ministry of Health [creator] (2014). Vital Events Deaths. V2. Population Data BC [publisher]. Data Extract. MOH (2014). [cited 2021 Mar 2].

[18] BC Cancer [creator] (2016). BC Cancer Registry Data. V2. Population Data BC [publisher]. Data Extract. BC Cancer (2014). [cited 2021 Mar 2].

[19] British Columbia Ministry of Health [creator] (2014). PharmaNet. V2. Popu- lation Data BC [publisher]. Data Extract. Data Stewardship Committee (2014). [cited 2021 Mar 2].

[20] Chronic Disease Information Working Group. BC Chronic Disease and Selected Procedure Case Definitions version 2017, last updated April 2019 [Internet]. British Columbia Ministry of Health; 2015. [cited 2021 Mar 2]. Available from: http://www.bccdc.ca/health-professionals/data-reports/chronic-disease-dashboard#Case%2D%2DDefinitions.

[21] Porta MS (2008). A dictionary of epidemiology. 5th ed. the international epidemiological association, editor.

[22] Taruc-Uy RL, Lynch SA (2013). Diagnosis and Treatment of Osteoarthritis. Primary Care - Clinics in Office Practice.

[23] Wang L, Panagiotoglou D, Min JE, DeBeck K, Milloy MJ, Kerr T (2016). Inability to access health and social services associated with mental health among people who inject drugs in a Canadian setting. Drug Alcohol Depend.

[24] Matsuzaki M, Vu QM, Gwadz M, Delaney JAC, Kuo I, Trejo MEP (2018). Perceived access and barriers to care among illicit drug users and hazardous drinkers: Findings from the Seek, Test, Treat, and Retain data harmonization initiative (STTR). BMC Public Health.

[25] Kim M, Chae KH, Chung YJ, Hwang H, Lee M, Kim HK, et al. The effect of the look-back period for estimating incidence using administrative data. BMC Health Serv Res. 2020;20(1) Available from: /pmc/articles/PMC7057623/. [cited 2021 Feb 25]. 10.1186/s12913-020-5016-y PMC7057623 32131818

[26] Abbas S, Ihle P, Köster I, Schubert I (2012). Estimation of disease incidence in claims data dependent on the length of follow-up: A methodological approach. Health Serv Res.

[27] Worthington JM, Gattellari M, Goumas C, Jalaludin B (2017). Methods in Neuroepidemiology differentiating incident from recurrent stroke using administrative data: the impact of varying lengths of look-Back periods on the risk of misclassification. Neuroepidemiology.

[28] Schmedt N, Khil L, Berger K, Riedel O (2017). Incidence of Multiple Sclerosis in Germany: A Cohort Study Applying Different Case Definitions Based on Claims Data. Neuroepidemiology.

[29] Griffiths RI, Omalley CD, Herbert RJ, Danese MD. Misclassification of incident conditions using claims data: Impact of varying the period used to exclude pre-existing disease. BMC Med Res Methodol. 2013;13(1) Available from: https://pubmed.ncbi.nlm.nih.gov/23496890/. [cited 2021 Feb 25]. 10.1186/1471-2288-13-32 PMC3602098 23496890

[30] Camplain R, Kucharska-Newton A, Cuthbertson CC, Wright JD, Alonso A, Heiss G (2017). Misclassification of incident hospitalized and outpatient heart failure in administrative claims data: the Atherosclerosis Risk in Communities (ARIC) study. Pharmacoepidemiol Drug Saf.

[31] Chronic Disease information working group (2015). BC Chronic Disease and selected procedure case definitions version 2016, last updated February 2018.

[32] Gilbertson DT, Bradbury BD, Wetmore JB, Weinhandl ED, Monda KL, Liu J (2015). Controlling confounding of treatment effects in administrative data in the presence of time-varying baseline confounders †.

[33] Corscadden L, Levesque JF, Lewis V, Strumpf E, Breton M, Russell G (2018). Factors associated with multiple barriers to access to primary care: An international analysis. Int J Equity Health.

