# Supplementary material for: The impact of lookback windows on the prevalence and incidence of chronic diseases among people living with HIV: an exploration in administrative health data in Canada
Source: BMC Med Res Methodol. 2022 Jan 6;22:1. doi: 10.1186/s12874-021-01448-x (PMC8734246; doi:10.1186/s12874-021-01448-x)
Supplement: Supplementary file 1 — Additional file 1. [file 12874_2021_1448_MOESM1_ESM.pdf]

Supplemental Materials

The impact of lookback windows on the prevalence and incidence of chronic diseases among people living with HIV: An exploration in administrative health data in Canada

Ni Gusti Ayu Nanditha[1,2], Xinzhe Dong[1], Taylor McLinden[1], Paul Sereda[1], Jacek Kopec[3,4], Robert S. Hogg[1,5], Julio S. G. Montaner[1,2], Viviane D. Lima[1,2*]

[1] British Columbia Centre for Excellence in HIV/AIDS, Vancouver, Canada

[2] Department of Medicine, Faculty of Medicine, University of British Columbia, Vancouver, Canada

[3] Arthritis Research Canada, Richmond, BC, Canada

[4] School of Population and Public Health, University of British Columbia, Vancouver, BC, Canada

[5] Faculty of Health Sciences, Simon Fraser University, Burnaby, BC, Canada

***Corresponding author**: Viviane D. Lima

BC Centre for Excellence in HIV/AIDS

608-1081 Burrard Street

Vancouver, BC | Canada V6Z 1Y6

Tel: 1-604-806-8796

E-mail: vlima@bccfe.ca

Table of Contents

Page 4 **Supplemental Table 1.** List of selected comorbidities and relevant case-finding algorithms.

Page 12 **Supplemental Table 2.** Prevalence of chronic diseases (as percentage, relative to a maximal 16-year lookback window) among people living with HIV and HIV-negative individuals in British Columbia, Canada for year 2012 across varying lookback windows, used to construct Figure 2.

Page 13 **Supplemental Table 3.** Incidence of chronic diseases (as percentage, relative to a maximal 16-year lookback window) among people living with HIV and HIV-negative individuals in British Columbia, Canada for year 2012 across varying lookback windows, used to construct Figure 3.

Page 14 **Supplemental Table 4.** Proportion of misclassified prevalent cases (as percentages, relative to the 10-year lookback window) of chronic diseases among people living with HIV and HIV-negative individuals in 2012 across varying lookback windows.

Page 15 **Supplemental Table 5.** Proportion of misclassified prevalent cases (as percentages, relative to the 13-year lookback window) of chronic diseases among people living with HIV and HIV-negative individuals in 2012 across varying lookback windows.

Page 16 **Supplemental Table 6.** Proportion of misclassified incident cases (as percentages, relative to the 10-year lookback window) of chronic diseases among people living with HIV and HIV-negative individuals in 2012 across varying lookback windows.

Page 17 **Supplemental Table 7.** Proportion of misclassified incident cases (as percentages, relative to the 13-year lookback window) of chronic diseases among people living with HIV and HIV-negative individuals in 2012 across varying lookback windows.

Page 18 **Supplemental Table 8.** Annual trends in prevalence of chronic diseases (as percentage) among people living with HIV in British Columbia from 2001 to 2012 using varying lookback windows, used to construct Figure 4.

Page 20 **Supplemental Table 9.** Annual trends in prevalence of chronic diseases (as percentage) among HIV-negative individuals in British Columbia from 2001 to 2012 using varying lookback windows, used to construct Supplemental Figure 2.

Page 22 **Supplemental Table 10.** Annual trends in incidence of chronic diseases (as percentage) among people living with HIV in British Columbia from 2001 to 2012 using varying lookback windows, used to construct Figure 5.

Page 24 **Supplemental Table 11.** Annual trends in incidence of chronic diseases (as percentage) among HIV-negative individuals in British Columbia from 2001 to 2012 using varying lookback windows, used to construct Supplemental Figure 3.

Page 26 **Supplemental Figure 1.** Flowchart outlining the derivation of the final 1:5 matched analytical sample of 5,151 PLWH and 25,755 HIV-negative individuals in British

Columba, Canada, who had administrative health records or public insurance registration since 1996 and were still alive in 2012

Page 28 **Supplemental Figure 2.** Annual trends in prevalence of chronic diseases among HIV-negative individuals in British Columbia from 2001 to 2012 using varying lookback windows.

Page 29 **Supplemental Figure 3.** Annual trends in incidence of chronic diseases among HIV-negative individuals in British Columbia from 2001 to 2012 using varying lookback windows.

Supplemental Table 1. List of selected comorbidities and relevant case-finding algorithms.

**Chronic Age-related Comorbidities**	**Case Definitions**	**Diagnostic, Procedure, and/or Drug Codes**	**References**
Alzheimer's and non-HIV-related dementia (ALZD)	Applicable to persons aged 40 years and older:<br><br>1 hospitalization with an ALZD diagnostic code<br><br>OR<br><br>3 physician visits, at least 30 days apart, in 2 years with ALZD diagnostic code(s)<br><br>OR<br><br>1 ALZD prescription drug	International Classification of Diseases Ninth Revision (ICD-9): 046.1, 290, 294, 331, 331.0, 331.1, 331.5<br><br>International Classification of Diseases Tenth Revision (ICD-10): G30, F00, F01, F02, F03<br><br>Drug Identification Numbers (DIN): 2232043, 2232044, 2242115, 2242116, 2242117, 2242118, 2244298, 2244299, 2244300, 2244302, 2245240, 2260638, 2266717, 2266725, 2266733, 2269457, 2269465, 2293021, 2293048, 2293056, 2295229, 2295237, 2295245, 2302845, 2302853, 2305984, 2305992, 2306018, 2306026, 2306034, 2306042, 2306050, 2306069, 2307685, 2307693, 2307707, 2307715, 2308169, 2308177, 2308185, 2308193, 2311283, 2311291, 2311305, 2311313, 2312492, 2312506, 2312514, 2312522, 2316943, 2316951, 2316978, 2320908, 2321130, 2321599, 2321602, 2322331, 2322358, 2324059, 2324067, 2324563, 2324571, 2324598, 2324601, 2328666, 2328682, 2332809, 2332817, 2332825, 2332833, 2333376, 2333384, 2333392, 2336715, 2336723, 2336731, 2336758, 2339439, 2339447, 2339455, 2340607, 2340615, 2344807, 2348950, 2349116, 2359472, 2359480, 2362260, 2362279, 2366487, 2367688, 2367696, 2375532, 2375729, 2375737, 2375745, 2375753, 2376334, 2377950, 2377969, 2377977, 2381508, 2381516, 2382830, 2386003, 2386011, 2386038, 2386046, 2392283, 2392291, 2392305, 2395584, 2395592, 2397595, 2397609, 2397617, 2397625, 2398370, 2398389, 2398397, 2398885, 2398893, 2400561, 2400588, 2401614, 2401622, 2401630, 2401649, 2402092, 2402106, 2402645, 2402653, 2404419, 2404427, 2406985, 2406993, 2407000, 2407019, 2408600, 2408619, 2409887, 2409895, 2412853, 2412861, 2412918, 2412934, 2413671, 2413698, 2416417, 2416425, 2416573, 2416581, 2416603, 2416948, 2416956, 2416999, 2417006, 2417014, 2417022, 2419238, 2419246, 2419254, 2419866, 2419874, 2420597, 2420600, 2420821, 2420848, 2420856, 2421364, 2421453, 2421461,	(1)

			2423413, 2423421, 2425157, 2425165, 2425173, 2425343, 2425351, 2425742, 2426293, 2426307, 2426943, 2426951, 2427273, 2427567, 2427575, 2427583, 2427591, 2428482, 2428490, 2430371, 2432684, 2432692, 2432803, 2439557, 2439565, 2443015, 2443023, 2443031, 2443082, 2446049, 2446669, 2446677, 2447002, 2447010	
Cardiovascular diseases (CVD)	Acute myocardial infarction (AMI)	1 hospitalization with an AMI diagnostic code	ICD-9: 410<br><br>ICD-10: I21	(1)
	Congestive heart failure (CHF)	1 hospitalization with a CHF diagnostic code<br><br>OR<br><br>2 physician visits in 1 year with CHF diagnostic code(s)	ICD-9: 428<br><br>ICD-10: I50	(1)
	Ischaemic heart disease (IHD)	Applicable to persons aged 20 years and older:<br><br>2 physician visits with Angina ICD-9 code 413 plus 1 prescription in 1 year<br><br>OR<br><br>1 specialist visit with Angina ICD-9 code 413 plus one prescription in 1 year<br><br>OR<br><br>2 physician visits with two ICD9 codes 410, 411, 412, 413, 414 in 1 year<br><br>OR<br><br>1 CABG, PCI/PCTA procedure code<br><br>OR<br><br>1 hospitalization with any IHD code(s)	ICD-9: 410, 411, 412, 413, 414<br><br>ICD-10: I20, I21, I22, I23, I24, I25<br><br>Canadian Classification of Health Interventions (CCI):<br>*CABG: 1IJ57LA, 1IJ57VS, 1IJ76<br>*PCI/PTCA: 1U50 ,1IJ57G<br><br>Canadian Classification of Diagnostic, Therapeutic, and Surgical Procedures (CCP):<br>*CABG:<br>4811, 4812, 4813, 4814, 4815, 4816, 4817, 4819<br>*PCI/PTCA:<br>4802, 4803<br><br>**Note: CABG: coronary artery bypass surgery; PCI/PTCA: percutaneous coronary intervention/ Percutaneous transluminal coronary angioplasty*	(1)
	Cerebrovascular accident (stroke/CVA)	Applicable to persons aged 20 years and older:<br><br>1 hospitalization with a CVA diagnostic code	ICD-9: 362.3, 430, 431, 433.x1, 434, 435, 436<br><br>ICD-10: H34.1, I60, I61, I63, I64	(1)

		Note: Cases occurring on the same day as a traumatic brain injury event are excluded.	*Exclusions: any traumatic brain injury*<br>ICD-9: 800, 801, 802, 803, 804, 850, 851, 852, 853, 854, V57.x<br><br>ICD-10: S02.x, S02.5, S06.x, Z50.x	
	Transient Ischemic Attack (mini stroke/TIA)	Looking at persons aged 20 and older:<br><br>1 hospitalization with a TIA diagnostic code<br><br>*Note: Cases occurring on the same day as a traumatic brain injury event are excluded. See exclusion codes for CVA above.*	ICD-9: 435<br><br>ICD-10: H34.0, G45.0, G45.1, G45.2, G45.3, G45.8, G45.9	(1)
Chronic obstructive pulmonary disease (COPD)		Looking at persons aged 35 and older:<br><br>1 hospitalization with a COPD diagnostic code<br><br>OR<br><br>2 physician visits in 1 year with COPD diagnostic code(s)	ICD-9: 491, 492, 496<br><br>ICD-10: J41, J42, J43, J44	(2)
Diabetes mellitus (DM)		1 hospitalization with a DM diagnostic code<br><br>OR<br><br>2 physician visits in 1 year with DM diagnostic code(s)<br><br>OR<br><br>2 or more insulin prescriptions in 1 year<br><br>OR<br><br>2 or more oral antihyperglycemic (not including metformin) prescriptions in 1 year<br><br>OR<br><br>1 insulin and 1 oral antihyperglycemic (including metformin) in 1 year<br><br>OR	ICD-9: 250<br><br>ICD-10: E10, E11, E12, E13, E14<br><br>Drug Identification Numbers (DIN): 5894, 6009, 12556, 12564, 12599, 12602, 12610, 13730, 13889, 15598, 21350, 21849, 24708, 24716, 93033, 156663, 156728, 178543, 209872, 209937, 237000, 244449, 271330, 274119, 274127, 275409, 275417, 275425, 312711, 312762, 314552, 377937, 399302, 420336, 430986, 431168, 446564, 446572, 446580, 446599, 446602, 446610, 454753, 480290, 480304, 513644, 514535, 514551, 539201, 539244, 542911, 542938, 542946, 546348, 552259, 552267, 552275, 554820, 586714, 586773, 587737, 612162, 612170, 612189, 612197, 612200, 612219, 612227, 612235, 612243, 612251, 612278, 612359, 614416, 628301, 632651, 632678, 632686, 632694, 644358, 646148, 648094, 650935, 720933, 720941, 723789, 733075, 765996, 773654, 795879, 808733, 808741, 889091, 889105, 889113, 889121, 999717, 1900927, 1900935, 1913654, 1913662, 1913670, 1913689, 1934066, 1934074, 1934082, 1934090, 1934104, 1934112, 1959212, 1959220, 1959239, 1959352, 1959360,	(1)

	2 metformin prescriptions and 1 physician visit with diabetes code(s) in 1 year.<br><br>*Note: Cases of suspected gestational diabetes in women aged 10-54 are not included by excluding hospitalizations, physician claims or prescriptions within the time period 120 days preceding or 180 days after hospital records containing birth-related diagnostic codes (see gestational diabetes exclusion codes).*	1962639, 1962647, 1962655, 1962663, 1985930, 1985949, 1985957, 1985965, 1985973, 1985981, 1986085, 1986791, 1986805, 1986813, 1986821, 1987534, 1987542, 1987828, 1987836, 2020734, 2020742, 2022230, 2022249, 2024217, 2024225, 2024233, 2024241, 2024268, 2024276, 2024284, 2024292, 2024306, 2024314, 2024322, 2024403, 2024446, 2025248, 2025256, 2045710, 2084341, 2085887, 2099233, 2147521, 2147548, 2148765, 2155850, 2162822, 2162849, 2167786, 2188902, 2190885, 2190893, 2220628, 2223562, 2224550, 2224569, 2224771, 2224798, 2226804, 2226812, 2228920, 2228939, 2229516, 2229517, 2229519, 2229595, 2229596, 2229656, 2229704, 2229705, 2229785, 2229994, 2230026, 2230027, 2230036, 2230037, 2230443, 2230444, 2230475, 2230670, 2230671, 2231058, 2231095, 2231096, 2231389, 2233562, 2233999, 2234513, 2234514, 2236543, 2236548, 2236733, 2236734, 2236985, 2236986, 2237531, 2238103, 2238469, 2238470, 2238471, 2238698, 2238827, 2239081, 2239214, 2239474, 2239475, 2239476, 2239924, 2239925, 2239926, 2240294, 2240295, 2240297, 2241111, 2241112, 2241113, 2241114, 2241283, 2241310, 2242095, 2242096, 2242572, 2242573, 2242574, 2242589, 2242726, 2242783, 2242793, 2242794, 2242931, 2242974, 2242987, 2244353, 2245247, 2245272, 2245273, 2245274, 2245397, 2245438, 2245439, 2245440, 2245689, 2246820, 2246821, 2246964, 2246965, 2247085, 2247086, 2247087, 2248008, 2248009, 2248210, 2248440, 2248441, 2248453, 2251930, 2252945, 2252953, 2254719, 2257726, 2257734, 2258781, 2258803, 2258811, 2265435, 2265443, 2265575, 2265583, 2268493, 2268507, 2269031, 2269058, 2269589, 2269597, 2269600, 2269619, 2271842, 2273101, 2273128, 2273136, 2273756, 2273764, 2273772, 2274248, 2274256, 2274264, 2274272, 2274914, 2274922, 2274930, 2275864, 2275872, 2276410, 2279061, 2279088, 2279126, 2279460, 2279479, 2279487, 2284545, 2284553, 2284782, 2284790, 2287072, 2294338, 2294346, 2294400, 2295377, 2295385, 2295393, 2297795, 2297906, 2297914, 2297922, 2298279, 2298287, 2298295, 2300451, 2301423, 2301431, 2301458, 2302861, 2302888, 2302896,

	2302942, 2302950, 2302977, 2303124, 2303132, 2303140, 2303442, 2303450, 2303469, 2303922, 2305062, 2306166, 2306174, 2306182, 2307170, 2307189, 2307197, 2307553, 2307561, 2307588, 2307634, 2307642, 2307650, 2307669, 2307677, 2307723, 2312050, 2312069, 2312077, 2313596, 2314894, 2314908, 2316544, 2320754, 2320762, 2320770, 2321475, 2321483, 2321491, 2326329, 2326337, 2326345, 2326477, 2326485, 2326493, 2331519, 2331527, 2333554, 2333856, 2333864, 2333872, 2334437, 2334445, 2336316, 2339110, 2339129, 2339587, 2339595, 2340763, 2340771, 2341522, 2341603, 2343606, 2343614, 2345366, 2345374, 2345382, 2345854, 2345862, 2348578, 2350459, 2350467, 2351056, 2351064, 2353377, 2353385, 2354144, 2354152, 2354160, 2354349, 2354357, 2354365, 2354926, 2354934, 2354942, 2355663, 2355671, 2355698, 2356422, 2357453, 2357461, 2357488, 2357887, 2357895, 2357909, 2357917, 2357925, 2361264, 2361272, 2361809, 2361817, 2363232, 2363240, 2363259, 2363518, 2363704, 2363712, 2364506, 2364514, 2365286, 2365294, 2365529, 2365537, 2366347, 2366355, 2366363, 2370921, 2373270, 2373289, 2373297, 2374013, 2374021, 2374048, 2374587, 2374595, 2375842, 2375850, 2375869, 2375877, 2377209, 2378043, 2378051, 2378116, 2378124, 2378620, 2378639, 2378841, 2378868, 2379767, 2379775, 2380196, 2380218, 2380722, 2380730, 2384906, 2384914, 2384922, 2385341, 2385368, 2388766, 2388774, 2388839, 2388847, 2389169, 2389177, 2389185, 2389290, 2389304, 2389312, 2391600, 2397307, 2403250, 2403269, 2403277, 2403366, 2403374, 2403382, 2403412, 2403420, 2403439, 2403447, 2405067, 2406020, 2406039, 2407124, 2408228, 2408236, 2409283, 2409291, 2412829, 2415089, 2415968, 2415976, 2415984, 2416786, 2416794, 2416808, 2417049, 2417057, 2417065, 2417189, 2417197, 2417200, 2417219, 2417227, 2417235, 2418002, 2418010, 2418029, 2419300, 2419319, 2419327, 2419335, 2419343, 2419351, 2421674, 2421682, 2421690, 2421828, 2421836, 2423286, 2424258, 2424266, 2424274, 2425483, 2425491, 2429764, 2429772, 2434121, 2434148, 2434156, 2435462, 2435470, 2437899,

			2438275, 2438283, 2438658, 2439328, 2439611, 2441829, 2443635, 2443643, 2443937, 2443945, 2444844, 2444852, 2444933, 2444941, 2446065, 2448599, 2448602, 2448610, 2449390, 2449404, 2449765, 2449935, 2449943, 2455404, 2455412, 2455420, 2455439, 2455447, 2455455, 2456575, 2456583, 2456591, 2456605, 2456613, 2456621, 22303140, 66123203<br><br>*Exclusions: gestational diabetes*<br>ICD-9: 641, 642, 643, 644, 645, 646, 647, 648, 650, 651, 652, 653, 654, 655, 656, 657, 658, 659, 660, 661, 662, 663, 664, 665, 666, 667, 668, 669, 670, 763, V27<br><br>ICD-10: O10, O11, O12, O13, O14, O15, O16, O21, O22, O23, O24, O25, O26, O28, O29, O30, O31, O32, O33, O34, O35, O36, O37, O40, O41, O42, O43, O44, O45, O46, O47, O48, O60, O61, O62, O63, O64, O65, O66, O67, O68, O69, O70, O71, O72, O73, O74, O75, O85, O86, O87, O88, O89, O90, O91, O92, O94, O95, O98, O99, Z37	
Hypertension (HTN)		Looking at persons aged 20 and older:<br><br>1 hospitalization with a hypertension diagnostic code<br><br>OR<br><br>2 physician visits in 2 years with hypertension diagnostic code(s)	ICD-9: 401, 402, 403, 404, 405<br><br>ICD-10: I10, I11, I12, I13, I15	(1)
Kidney diseases (KID)	Chronic kidney disease (CKD)	1 hospitalization with a CKD diagnostic code<br><br>OR<br><br>2 physician visits in 1 year with CKD diagnostic code(s)	ICD-9: 581, 582, 583, 585, 586, 587, 589<br><br>ICD-10: N01, N03, N04, N05, N06, N07, N18, N19, N26, N27	(1)
	Dialysis	9 or more physician visits within 90 days with dialysis fee item code(s)	Fee Codes: 00308, 00323, 00350, 00351, 00352, 00355, 00356, 00358, 00359, 00361, 00390, 33708, 33723, 33750, 33751, 33752, 33755, 33756, 33758, 33759, 33761, 33790	(1)
	Kidney transplant	1 hospitalization with procedure code(s) for kidney transplant	CCI: 675<br><br>CCP: 1PC85	(1)
Liver diseases (LVR)	Cirrhosis	1 hospitalization with a cirrhosis diagnostic code<br><br>OR	ICD-9 for physician visit: 571<br><br>ICD-9 for hospitalization: 456.1, 571.2, 571.5	(3)

		2 physician visits with cirrhosis diagnostic code(s) in 1 year	ICD-10: I85.9, I98.2, K70.3, K71.7, K74.6	
	Decompensated cirrhosis	1 hospitalization with a decompensated cirrhosis diagnostic code<br><br>OR<br><br>2 physician visits with a decompensated cirrhosis diagnostic code(s) in 1 year	ICD-9: 456.0, 456.2, 572.2, 572.3, 572.4, 782.4, 789.5<br><br>ICD-10: I85.0, I86.4, I98.20, I98.3, K72.1, K72.9, K76.6, K76.7, R17, R18	(3)
	Chronic liver disease (CLD)	1 hospitalization with a CLD diagnostic code<br><br>OR<br><br>2 physician visits with CLD diagnostic code(s) in 1 year	ICD-9: 070.2, 070.3, 070.41, 070.44, 070.51, 070.54, 070.7, 275.0, 573.3<br><br>ICD-10: B16, B17.10, B17.11, B18.0, B18.1, B18.2, B19.10, B19.11, B19.20, B19.21, E83.11, K71.6, K75.9<br><br>*Note: We modified the case definition to "2 physician visits/year", as opposed to "1 physician visit", to allow for a confirmatory visit. We have also removed diagnostic codes that overlap with those in cirrhosis and decompensated cirrhosis.*	(4)
Osteoarthritis (OA)		1 hospitalization with an OA diagnostic code<br><br>OR<br><br>2 physician visits with OA diagnostic code(s) in 1 year	ICD-9: 715<br><br>ICD-10: M15, M16, M17, M18, M19	(1)

References

1. Chronic Disease Information Working Group. BC Chronic Disease and Selected Procedure Case Definitions version 2016, last updated February 2018. 2015;
2. Chronic Disease Information Working Group. BC Chronic Disease and Selected Procedure Case Definitions version 2017, last updated April 2019. 2015;(http://www.bccdc.ca/health-professionals/data-reports/chronic-disease-dashboard#Case--Definitions)
3. Lapointe-Shaw L, Georgie F, Carlone D, et al. Identifying cirrhosis, decompensated cirrhosis and hepatocellular carcinoma in health administrative data: A validation study. *PLoS One* [electronic

article]. 2018;13(8):e0201120. (https://dx.plos.org/10.1371/journal.pone.0201120). (Accessed August 9, 2019)

4. Roberts HW, Utuama OA, Klevens M, et al. The Contribution of Viral Hepatitis to the Burden of Chronic Liver Disease in the United States. *Am. J. Gastroenterol.* [electronic article]. 2014;109(3):387–393. (http://insights.ovid.com/crossref?an=00000434-201403000-00015). (Accessed August 14, 2019)

Supplemental Table 2. Number of prevalent cases and prevalence of chronic diseases (as a percentage, relative to a maximal 16-year lookback window) among people living with HIV and HIV-negative individuals in British Columbia, Canada for year 2012 across varying lookback windows, used to construct Figure 2.

Chronic Disease	HIV Status	Lookback Window																
		0	1	2	3	4	5	6	7	8	9	10	11	12	13	14	15	16
Alzheimer's/ and/or Dementia	PLWH	33 (0.7)	43 (1.0)	53 (1.2)	62 (1.4)	71 (1.6)	77 (1.7)	88 (2.0)	93 (2.1)	99 (2.2)	103 (2.3)	107 (2.4)	109 (2.5)	112 (2.5)	112 (2.5)	115 (2.6)	118 (2.7)	119 (2.7)
	HIV-Neg	40 (0.2)	52 (0.2)	57 (0.3)	59 (0.3)	69 (0.3)	71 (0.3)	71 (0.3)	74 (0.3)	77 (0.3)	80 (0.4)	80 (0.4)	85 (0.4)	86 (0.4)	87 (0.4)	91 (0.4)	92 (0.4)	92 (0.4)
COPD	PLWH	151 (3.2)	203 (4.3)	251 (5.3)	275 (5.8)	293 (6.2)	303 (6.4)	316 (6.6)	324 (6.8)	336 (7.1)	343 (7.2)	349 (7.3)	350 (7.4)	356 (7.5)	363 (7.6)	366 (7.7)	371 (7.8)	374 (7.9)
	HIV-Neg	247 (1.0)	353 (1.5)	429 (1.8)	472 (2)	506 (2.1)	538 (2.3)	556 (2.3)	577 (2.4)	595 (2.5)	608 (2.6)	629 (2.6)	648 (2.7)	662 (2.8)	677 (2.8)	685 (2.9)	689 (2.9)	697 (2.9)
Cardiovascular Diseases	PLWH	88 (1.7)	145 (2.8)	186 (3.6)	220 (4.3)	256 (5)	288 (5.6)	310 (6)	319 (6.2)	346 (6.7)	355 (6.9)	366 (7.1)	376 (7.3)	384 (7.5)	389 (7.6)	397 (7.7)	409 (7.9)	414 (8.0)
	HIV-Neg	314 (1.2)	511 (2.0)	649 (2.5)	763 (3)	876 (3.4)	966 (3.8)	1038 (4)	1114 (4.3)	1187 (4.6)	1236 (4.8)	1281 (5)	1317 (5.1)	1351 (5.2)	1375 (5.3)	1397 (5.4)	1414 (5.5)	1431 (5.6)
Diabetes Mellitus	PLWH	364 (7.1)	423 (8.2)	456 (8.9)	483 (9.4)	499 (9.7)	518 (10.1)	527 (10.2)	538 (10.4)	552 (10.7)	564 (10.9)	575 (11.2)	593 (11.5)	594 (11.5)	598 (11.6)	598 (11.6)	600 (11.6)	601 (11.7)
	HIV-Neg	1825 (7.1)	2053 (8.0)	2185 (8.5)	2274 (8.8)	2348 (9.1)	2413 (9.4)	2457 (9.5)	2480 (9.6)	2501 (9.7)	2516 (9.8)	2540 (9.9)	2555 (9.9)	2569 (10.0)	2576 (10.0)	2589 (10.1)	2594 (10.1)	2599 (10.1)
Hypertension	PLWH	387 (7.5)	577 (11.2)	679 (13.2)	750 (14.6)	814 (15.8)	874 (17)	903 (17.5)	928 (18)	942 (18.3)	964 (18.7)	975 (18.9)	982 (19.1)	995 (19.3)	1005 (19.5)	1015 (19.7)	1024 (19.9)	1026 (19.9)
	HIV-Neg	2817 (10.9)	3591 (13.9)	4071 (15.8)	4408 (17.1)	4678 (18.2)	4876 (18.9)	5043 (19.6)	5180 (20.1)	5312 (20.6)	5404 (21.0)	5487 (21.3)	5548 (21.5)	5594 (21.7)	5639 (21.9)	5670 (22)	5702 (22.1)	5726 (22.2)
Kidney Diseases	PLWH	120 (2.3)	168 (3.3)	218 (4.2)	267 (5.2)	326 (6.3)	371 (7.2)	419 (8.1)	447 (8.7)	482 (9.4)	502 (9.7)	515 (10)	526 (10.2)	540 (10.5)	560 (10.9)	577 (11.2)	590 (11.5)	597 (11.6)
	HIV-Neg	153 (0.6)	210 (0.8)	238 (0.9)	278 (1.1)	300 (1.2)	316 (1.2)	329 (1.3)	343 (1.3)	352 (1.4)	365 (1.4)	369 (1.4)	373 (1.4)	383 (1.5)	387 (1.5)	393 (1.5)	402 (1.6)	404 (1.6)
Liver Diseases	PLWH	212 (4.1)	364 (7.1)	516 (10)	627 (12.2)	699 (13.6)	764 (14.8)	842 (16.3)	916 (17.8)	975 (18.9)	1039 (20.2)	1079 (20.9)	1116 (21.7)	1134 (22)	1161 (22.5)	1180 (22.9)	1198 (23.3)	1209 (23.5)
	HIV-Neg	126 (0.5)	188 (0.7)	236 (0.9)	275 (1.1)	303 (1.2)	333 (1.3)	359 (1.4)	377 (1.5)	405 (1.6)	426 (1.7)	447 (1.7)	471 (1.8)	489 (1.9)	504 (2.0)	521 (2.0)	534 (2.1)	537 (2.1)
Osteoarthritis	PLWH	82 (1.6)	124 (2.4)	189 (3.7)	224 (4.3)	258 (5)	295 (5.7)	323 (6.3)	338 (6.6)	359 (7.0)	380 (7.4)	393 (7.6)	404 (7.8)	420 (8.2)	438 (8.5)	456 (8.9)	466 (9)	478 (9.3)
	HIV-Neg	474 (1.8)	742 (2.9)	950 (3.7)	1152 (4.5)	1320 (5.1)	1446 (5.6)	1560 (6.1)	1663 (6.5)	1758 (6.8)	1819 (7.1)	1889 (7.3)	1946 (7.6)	2000 (7.8)	2053 (8.0)	2095 (8.1)	2145 (8.3)	2186 (8.5)

Note: PLWH: people living with HIV; HIV-Neg: HIV-negative individuals; COPD: Chronic Obstructive Pulmonary Diseases.

Supplemental Table 3. Number of incident cases and incidence of chronic diseases (as a percentage, relative to a maximal 16-year lookback window) among people living with HIV and HIV-negative individuals in British Columbia, Canada for year 2012 across varying lookback windows, used to construct Figure 3.

Chronic Disease	HIV Status	Lookback Window																
		0	1	2	3	4	5	6	7	8	9	10	11	12	13	14	15	16
Alzheimer's/ and/or Dementia	PLWH	33 (0.7)	17 (0.4)	15 (0.3)	14 (0.3)	14 (0.3)	13 (0.3)	13 (0.3)	13 (0.3)	13 (0.3)	13 (0.3)	13 (0.3)	13 (0.3)	13 (0.3)	13 (0.3)	13 (0.3)	13 (0.3)	13 (0.3)
	HIV-Neg	40 (0.2)	14 (0.1)	12 (0.1)	12 (0.1)	12 (0.1)	12 (0.1)	12 (0.1)	12 (0.1)	12 (0.1)	12 (0.1)	12 (0.1)	12 (0.1)	12 (0.1)	12 (0.1)	11 (0.1)	11 (0.1)	11 (0.1)
COPD	PLWH	151 (3.2)	74 (1.6)	56 (1.2)	51 (1.1)	48 (1.1)	47 (1.0)	46 (1.0)	46 (1.0)	45 (1.0)	45 (1.0)	45 (1.0)	45 (1.0)	45 (1.0)	45 (1.0)	45 (1.0)	45 (1.0)	45 (1.0)
	HIV-Neg	247 (1.0)	103 (0.4)	92 (0.4)	89 (0.4)	88 (0.4)	85 (0.4)	84 (0.4)	84 (0.4)	84 (0.4)	82 (0.4)	81 (0.3)	79 (0.3)	79 (0.3)	78 (0.3)	78 (0.3)	78 (0.3)	78 (0.3)
Cardiovascular Diseases	PLWH	88 (1.7)	59 (1.2)	57 (1.1)	53 (1.1)	50 (1.0)	48 (1.0)	47 (1.0)	46 (0.9)	45 (0.9)	45 (0.9)	45 (0.9)	44 (0.9)	44 (0.9)	44 (0.9)	44 (0.9)	44 (0.9)	44 (0.9)
	HIV-Neg	314 (1.2)	210 (0.8)	190 (0.8)	175 (0.7)	168 (0.7)	163 (0.7)	157 (0.6)	154 (0.6)	151 (0.6)	150 (0.6)	148 (0.6)	145 (0.6)	140 (0.6)	139 (0.6)	139 (0.6)	137 (0.6)	137 (0.6)
Diabetes Mellitus	PLWH	364 (7.1)	65 (1.4)	56 (1.2)	51 (1.1)	47 (1)	43 (0.9)	39 (0.8)	38 (0.8)	38 (0.8)	37 (0.8)	37 (0.8)	37 (0.8)	37 (0.8)	37 (0.8)	37 (0.8)	37 (0.8)	37 (0.8)
	HIV-Neg	1825 (7.1)	249 (1.0)	205 (0.9)	193 (0.8)	185 (0.8)	179 (0.8)	174 (0.7)	172 (0.7)	169 (0.7)	168 (0.7)	165 (0.7)	165 (0.7)	164 (0.7)	164 (0.7)	164 (0.7)	164 (0.7)	164 (0.7)
Hypertension	PLWH	387 (7.5)	155 (3.3)	119 (2.6)	101 (2.2)	91 (2.1)	87 (2.0)	83 (1.9)	80 (1.9)	76 (1.8)	75 (1.8)	74 (1.7)	74 (1.7)	73 (1.7)	71 (1.7)	71 (1.7)	70 (1.7)	70 (1.7)
	HIV-Neg	2817 (10.9)	727 (3.2)	553 (2.5)	471 (2.2)	430 (2.0)	404 (1.9)	389 (1.8)	374 (1.8)	356 (1.7)	351 (1.7)	346 (1.7)	340 (1.7)	336 (1.6)	336 (1.6)	332 (1.6)	331 (1.6)	330 (1.6)
Kidney Diseases	PLWH	120 (2.3)	54 (1.1)	46 (0.9)	37 (0.8)	35 (0.7)	35 (0.7)	34 (0.7)	34 (0.7)	33 (0.7)	33 (0.7)	33 (0.7)	33 (0.7)	33 (0.7)	33 (0.7)	32 (0.7)	32 (0.7)	32 (0.7)
	HIV-Neg	153 (0.6)	64 (0.2)	61 (0.2)	58 (0.2)	57 (0.2)	55 (0.2)	55 (0.2)	54 (0.2)	53 (0.2)	53 (0.2)	53 (0.2)	53 (0.2)	53 (0.2)	53 (0.2)	53 (0.2)	53 (0.2)	53 (0.2)
Liver Diseases	PLWH	212 (4.1)	126 (2.6)	100 (2.1)	83 (1.8)	79 (1.7)	75 (1.7)	70 (1.6)	69 (1.6)	67 (1.6)	65 (1.6)	62 (1.5)	62 (1.5)	62 (1.5)	61 (1.5)	61 (1.5)	61 (1.5)	59 (1.5)
	HIV-Neg	126 (0.5)	77 (0.3)	73 (0.3)	69 (0.3)	67 (0.3)	63 (0.2)	63 (0.2)	63 (0.2)	63 (0.2)	63 (0.2)	62 (0.2)	61 (0.2)	61 (0.2)	61 (0.2)	61 (0.2)	61 (0.2)	61 (0.2)
Osteoarthritis	PLWH	82 (1.6)	46 (0.9)	44 (0.9)	43 (0.9)	41 (0.8)	40 (0.8)	39 (0.8)	39 (0.8)	38 (0.8)	36 (0.7)	36 (0.8)	36 (0.8)	36 (0.8)	34 (0.7)	33 (0.7)	32 (0.7)	32 (0.7)
	HIV-Neg	474 (1.8)	279 (1.1)	246 (1.0)	230 (0.9)	215 (0.9)	202 (0.8)	196 (0.8)	190 (0.8)	187 (0.8)	187 (0.8)	185 (0.8)	184 (0.8)	184 (0.8)	182 (0.8)	180 (0.8)	179 (0.8)	178 (0.7)

Note: PLWH: people living with HIV; HIV-Neg: HIV-negative individuals; COPD: Chronic Obstructive Pulmonary Diseases

Supplemental Table 4. Proportion of misclassified prevalent cases (as percentages, relative to the 10-year lookback window) of chronic diseases among people living with HIV and HIV-negative individuals in 2012 across varying lookback windows.

Chronic Disease	HIV Status	Lookback Window										
		0	1	2	3	4	5	6	7	8	9	10
Alzheimer's and/or Dementia	PLWH	69.2	59.8	50.5	42.1	33.6	28.0	17.8	13.1	7.5	3.7	Ref
	HIV-Neg	50.0	35.0	28.8	26.3	13.8	11.3	11.3	7.5	3.8	0.0	Ref
COPD	PLWH	56.7	41.8	28.1	21.2	16.0	13.2	9.5	7.2	3.7	1.7	Ref
	HIV-Neg	60.7	43.9	31.8	25.0	19.6	14.5	11.6	8.3	5.4	3.3	Ref
Cardiovascular Diseases	PLWH	76.0	60.4	49.2	39.9	30.1	21.3	15.3	12.8	5.5	3.0	Ref
	HIV-Neg	75.5	60.1	49.3	40.4	31.6	24.6	19.0	13.0	7.3	3.5	Ref
Diabetes Mellitus	PLWH	36.7	26.4	20.7	16.0	13.2	9.9	8.3	6.4	4.0	1.9	Ref
	HIV-Neg	28.1	19.2	14.0	10.5	7.6	5.0	3.3	2.4	1.5	0.9	Ref
Hypertension	PLWH	60.3	40.8	30.4	23.1	16.5	10.4	7.4	4.8	3.4	1.1	Ref
	HIV-Neg	48.7	34.6	25.8	19.7	14.7	11.1	8.1	5.6	3.2	1.5	Ref
Kidney Diseases	PLWH	76.7	67.4	57.7	48.2	36.7	28.0	18.6	13.2	6.4	2.5	Ref
	HIV-Neg	58.5	43.1	35.5	24.7	18.7	14.4	10.8	7.0	4.6	1.1	Ref
Liver Diseases	PLWH	80.4	66.3	52.2	41.9	35.2	29.2	22.0	15.1	9.6	3.7	Ref
	HIV-Neg	71.8	57.9	47.2	38.5	32.2	25.5	19.7	15.7	9.4	4.7	Ref
Osteoarthritis	PLWH	79.1	68.4	51.9	43.0	34.4	24.9	17.8	14.0	8.7	3.3	Ref
	HIV-Neg	74.9	60.7	49.7	39.0	30.1	23.5	17.4	12.0	6.9	3.7	Ref

Note: PLWH: people living with HIV; HIV-Neg: HIV-negative individuals; COPD: Chronic Obstructive Pulmonary Diseases; Ref: reference. Lightest, medium and darkest shade represent proportion of misclassification <10%, <20% and <30%, respectively. Proportion of misclassified prevalent cases was proportion of prevalent cases (i.e., identified using 10-year lookback window) not captured by a shorter lookback window.

Supplemental Table 5. Proportion of misclassified prevalent cases (as percentages, relative to the 13-year lookback window) of chronic diseases among people living with HIV and HIV-negative individuals in 2012 across varying lookback windows.

Chronic Disease	HIV Status	Lookback Window													
		0	1	2	3	4	5	6	7	8	9	10	11	12	13
Alzheimer's and/or Dementia	PLWH	70.5	61.6	52.7	44.6	36.6	31.3	21.4	17.0	11.6	8.0	4.5	2.7	0.0	Ref
	HIV-Neg	54.0	40.2	34.5	32.2	20.7	18.4	18.4	14.9	11.5	8.0	8.0	2.3	1.1	Ref
COPD	PLWH	58.4	44.1	30.9	24.2	19.3	16.5	12.9	10.7	7.4	5.5	3.9	3.6	1.9	Ref
	HIV-Neg	63.5	47.9	36.6	30.3	25.3	20.5	17.9	14.8	12.1	10.2	7.1	4.3	2.2	Ref
Cardiovascular Diseases	PLWH	77.4	62.7	52.2	43.4	34.2	26.0	20.3	18.0	11.1	8.7	5.9	3.3	1.3	Ref
	HIV-Neg	77.2	62.8	52.8	44.5	36.3	29.7	24.5	19.0	13.7	10.1	6.8	4.2	1.7	Ref
Diabetes Mellitus	PLWH	39.1	29.3	23.7	19.2	16.6	13.4	11.9	10.0	7.7	5.7	3.8	0.8	0.7	Ref
	HIV-Neg	29.2	20.3	15.2	11.7	8.9	6.3	4.6	3.7	2.9	2.3	1.4	0.8	0.3	Ref
Hypertension	PLWH	61.5	42.6	32.4	25.4	19.0	13.0	10.1	7.7	6.3	4.1	3.0	2.3	1.0	Ref
	HIV-Neg	50.0	36.3	27.8	21.8	17.0	13.5	10.6	8.1	5.8	4.2	2.7	1.6	0.8	Ref
Kidney Diseases	PLWH	78.6	70.0	61.1	52.3	41.8	33.8	25.2	20.2	13.9	10.4	8.0	6.1	3.6	Ref
	HIV-Neg	60.5	45.7	38.5	28.2	22.5	18.3	15.0	11.4	9.0	5.7	4.7	3.6	1.0	Ref
Liver Diseases	PLWH	81.7	68.6	55.6	46.0	39.8	34.2	27.5	21.1	16.0	10.5	7.1	3.9	2.3	Ref
	HIV-Neg	75.0	62.7	53.2	45.4	39.9	33.9	28.8	25.2	19.6	15.5	11.3	6.5	3.0	Ref
Osteoarthritis	PLWH	81.3	71.7	56.8	48.9	41.1	32.6	26.3	22.8	18.0	13.2	10.3	7.8	4.1	Ref
	HIV-Neg	76.9	63.9	53.7	43.9	35.7	29.6	24.0	19.0	14.4	11.4	8.0	5.2	2.6	Ref

Note: PLWH: people living with HIV; HIV-Neg: HIV-negative individuals; COPD: Chronic Obstructive Pulmonary Diseases; Ref: reference. Lightest, medium and darkest shade represent proportion of misclassification <10%, <20% and <30%, respectively. Proportion of misclassified prevalent cases was proportion of prevalent cases (i.e., identified using 13-year lookback window) not captured by a shorter lookback window.

Supplemental Table 6. Proportion of misclassified incident cases (as percentages, relative to the 10year lookback window) of chronic diseases among people living with HIV and HIV-negative individuals in 2012 across varying lookback windows.

Chronic Disease	HIV Status	Lookback Window										
		0	1	2	3	4	5	6	7	8	9	10
Alzheimer's and/or Dementia	PLWH	60.6	23.5	13.3	7.1	7.1	0.0	0.0	0.0	0.0	0.0	Ref
	HIV-Neg	70.0	14.3	0.0	0.0	0.0	0.0	0.0	0.0	0.0	0.0	Ref
COPD	PLWH	70.2	39.2	19.6	11.8	6.3	4.3	2.2	2.2	0.0	0.0	Ref
	HIV-Neg	67.2	21.4	12.0	9.0	8.0	4.7	3.6	3.6	3.6	1.2	Ref
Cardiovascular Diseases	PLWH	48.9	23.7	21.1	15.1	10.0	6.3	4.3	2.2	0.0	0.0	Ref
	HIV-Neg	52.9	29.5	22.1	15.4	11.9	9.2	5.7	3.9	2.0	1.3	Ref
Diabetes Mellitus	PLWH	89.8	43.1	33.9	27.5	21.3	14.0	5.1	2.6	2.6	0.0	Ref
	HIV-Neg	91.0	33.7	19.5	14.5	10.8	7.8	5.2	4.1	2.4	1.8	Ref
Hypertension	PLWH	80.9	52.3	37.8	26.7	18.7	14.9	10.8	7.5	2.6	1.3	Ref
	HIV-Neg	87.7	52.4	37.4	26.5	19.5	14.4	11.1	7.5	2.8	1.4	Ref
Kidney Diseases	PLWH	72.5	38.9	28.3	10.8	5.7	5.7	2.9	2.9	0.0	0.0	Ref
	HIV-Neg	65.4	17.2	13.1	8.6	7.0	3.6	3.6	1.9	0.0	0.0	Ref
Liver Diseases	PLWH	70.8	50.8	38.0	25.3	21.5	17.3	11.4	10.1	7.5	4.6	Ref
	HIV-Neg	50.8	19.5	15.1	10.1	7.5	1.6	1.6	1.6	1.6	1.6	Ref
Osteoarthritis	PLWH	56.1	21.7	18.2	16.3	12.2	10.0	7.7	7.7	5.3	0.0	Ref
	HIV-Neg	61.0	33.7	24.8	19.6	14.0	8.4	5.6	2.6	1.1	1.1	Ref

Note: PLWH: people living with HIV; HIV-Neg: HIV-negative individuals; COPD: Chronic Obstructive Pulmonary Diseases; Ref: reference. Lightest, medium and darkest shade represent proportion of misclassification <10%, <20% and <30%, respectively. Proportion of misclassified incident cases was proportion of incident cases as captured by a shorter lookback window that, in fact, were prevalent (i.e., when identified using 16-year lookback window).

Supplemental Table 7. Proportion of misclassified incident cases (as percentages, relative to the 13-year lookback window) of chronic diseases among people living with HIV and HIV-negative individuals in 2012 across varying lookback windows.

Chronic Disease	HIV Status	Lookback Window													
		0	1	2	3	4	5	6	7	8	9	10	11	12	13
Alzheimer's and/or Dementia	PLWH	60.6	23.5	13.3	7.1	7.1	0.0	0.0	0.0	0.0	0.0	0.0	0.0	0.0	Ref
	HIV-Neg	70.0	14.3	0.0	0.0	0.0	0.0	0.0	0.0	0.0	0.0	0.0	0.0	0.0	Ref
COPD	PLWH	70.2	39.2	19.6	11.8	6.3	4.3	2.2	2.2	0.0	0.0	0.0	0.0	0.0	Ref
	HIV-Neg	68.4	24.3	15.2	12.4	11.4	8.2	7.1	7.1	7.1	4.9	3.7	1.3	1.3	Ref
Cardiovascular Diseases	PLWH	50.0	25.4	22.8	17.0	12.0	8.3	6.4	4.3	2.2	2.2	2.2	0.0	0.0	Ref
	HIV-Neg	55.7	33.8	26.8	20.6	17.3	14.7	11.5	9.7	7.9	7.3	6.1	4.1	0.7	Ref
Diabetes Mellitus	PLWH	89.8	43.1	33.9	27.5	21.3	14.0	5.1	2.6	2.6	0.0	0.0	0.0	0.0	Ref
	HIV-Neg	91.0	34.1	20.0	15.0	11.4	8.4	5.7	4.7	3.0	2.4	0.6	0.6	0.0	Ref
Hypertension	PLWH	81.7	54.2	40.3	29.7	22.0	18.4	14.5	11.3	6.6	5.3	4.1	4.1	2.7	Ref
	HIV-Neg	88.1	53.8	39.2	28.7	21.9	16.8	13.6	10.2	5.6	4.3	2.9	1.2	0.0	Ref
Kidney Diseases	PLWH	72.5	38.9	28.3	10.8	5.7	5.7	2.9	2.9	0.0	0.0	0.0	0.0	0.0	Ref
	HIV-Neg	65.4	17.2	13.1	8.6	7.0	3.6	3.6	1.9	0.0	0.0	0.0	0.0	0.0	Ref
Liver Diseases	PLWH	71.2	51.6	39.0	26.5	22.8	18.7	12.9	11.6	9.0	6.2	1.6	1.6	1.6	Ref
	HIV-Neg	51.6	20.8	16.4	11.6	9.0	3.2	3.2	3.2	3.2	3.2	1.6	0.0	0.0	Ref
Osteoarthritis	PLWH	58.5	26.1	22.7	20.9	17.1	15.0	12.8	12.8	10.5	5.6	5.6	5.6	5.6	Ref
	HIV-Neg	61.6	34.8	26.0	20.9	15.3	9.9	7.1	4.2	2.7	2.7	1.6	1.1	1.1	Ref

Note: PLWH: people living with HIV; HIV-Neg: HIV-negative individuals; COPD: Chronic Obstructive Pulmonary Diseases; Ref: reference. Lightest, medium and darkest shade represent proportion of misclassification <10%, <20% and <30%, respectively. Proportion of misclassified incident cases was proportion of incident cases as captured by a shorter lookback window that, in fact, were prevalent (i.e., when identified using 13-year lookback window).

Supplemental Table 8. Annual trends in prevalence of chronic diseases (as percentage) among people living with HIV in British Columbia from 2001 to 2012 using varying lookback windows, used to construct Figure 4.

Chronic Disease	Year	Lookback Window																
		0	1	2	3	4	5	6	7	8	9	10	11	12	13	14	15	16
Alzheimer's/ and/or Dementia	2001	0.2	0.4	0.4	0.5	0.6	0.7											
	2002	0.4	0.4	0.6	0.6	0.7	0.8	0.8										
	2003	0.3	0.5	0.6	0.7	0.7	0.8	0.9	1.0									
	2004	0.4	0.5	0.7	0.8	0.9	0.9	1.0	1.0	1.1								
	2005	0.3	0.5	0.6	0.8	0.9	1.0	1.0	1.0	1.1	1.2							
	2006	0.6	0.8	1.0	1.1	1.2	1.3	1.3	1.3	1.4	1.5	1.5						
	2007	0.3	0.7	0.9	1.0	1.1	1.3	1.3	1.4	1.4	1.5	1.6	1.6					
	2008	0.5	0.7	1.0	1.2	1.3	1.4	1.6	1.6	1.7	1.7	1.8	1.8	1.9				
	2009	0.7	0.9	1.1	1.4	1.5	1.7	1.8	1.9	1.9	2.0	2.0	2.1	2.1	2.2			
	2010	0.6	0.9	1.1	1.3	1.6	1.7	1.8	1.9	2.0	2.1	2.1	2.1	2.2	2.3	2.3		
	2011	0.6	0.9	1.1	1.3	1.5	1.7	1.9	2.0	2.1	2.2	2.2	2.3	2.3	2.4	2.4	2.5	
	2012	0.7	1.0	1.2	1.4	1.6	1.7	2.0	2.1	2.2	2.3	2.4	2.5	2.5	2.5	2.6	2.7	2.7
COPD	2001	0.1	0.4	0.7	0.8	1.0	1.1											
	2002	0.3	0.4	0.6	0.9	1.0	1.2	1.3										
	2003	0.5	0.7	0.8	1.0	1.3	1.4	1.5	1.6									
	2004	0.7	1.1	1.2	1.3	1.5	1.7	1.8	2.0	2.0								
	2005	0.6	1.1	1.4	1.6	1.6	1.8	2.0	2.1	2.2	2.3							
	2006	0.8	1.2	1.6	1.8	2.0	2.0	2.2	2.4	2.5	2.6	2.7						
	2007	1.0	1.4	1.7	2.1	2.3	2.5	2.5	2.7	2.9	2.9	3.1	3.2					
	2008	1.4	1.8	2.2	2.5	2.8	3.0	3.2	3.2	3.3	3.5	3.6	3.7	3.8				
	2009	1.9	2.8	3.1	3.5	3.8	4.1	4.3	4.4	4.4	4.5	4.7	4.8	4.9	5.0			
	2010	2.6	3.5	4.1	4.4	4.7	4.9	5.2	5.4	5.5	5.5	5.7	5.8	5.9	6.0	6.1		
	2011	2.7	4.1	4.8	5.2	5.4	5.7	5.9	6.2	6.3	6.5	6.5	6.6	6.8	6.8	6.9	7.0	
	2012	3.2	4.3	5.3	5.8	6.2	6.4	6.6	6.8	7.1	7.2	7.3	7.4	7.5	7.6	7.7	7.8	7.9
Cardiovascular Diseases	2001	0.6	1.0	1.2	1.5	1.7	1.9											
	2002	0.7	1.1	1.5	1.7	1.9	2.2	2.3										
	2003	0.6	1.1	1.5	1.8	1.9	2.2	2.4	2.5									
	2004	0.9	1.3	1.7	2.0	2.3	2.5	2.7	3.0	3.0								
	2005	0.7	1.5	1.8	2.2	2.5	2.8	2.9	3.1	3.4	3.5							
	2006	1.1	1.5	2.2	2.5	2.9	3.2	3.5	3.6	3.8	4.0	4.1						
	2007	1.2	2.0	2.3	3.0	3.3	3.6	3.8	4.1	4.2	4.4	4.6	4.7					
	2008	1.2	2.1	2.7	3.0	3.7	3.9	4.2	4.4	4.6	4.8	5.0	5.2	5.3				
	2009	1.3	2.2	2.9	3.5	3.7	4.3	4.6	4.8	5.0	5.3	5.4	5.6	5.8	5.9			
	2010	1.4	2.2	3.0	3.7	4.2	4.4	5.0	5.2	5.5	5.7	5.8	6.0	6.1	6.4	6.5		
	2011	1.7	2.5	3.2	4.0	4.7	5.1	5.3	5.8	6.0	6.2	6.4	6.6	6.7	6.9	7.1	7.2	
	2012	1.7	2.8	3.6	4.3	5.0	5.6	6.0	6.2	6.7	6.9	7.1	7.3	7.5	7.6	7.7	7.9	8.0
Diabetes Mellitus	2001	2.5	2.7	2.8	2.9	3.0	3.0											
	2002	2.6	3.3	3.5	3.6	3.6	3.6	3.6										
	2003	2.9	3.3	3.9	4.0	4.1	4.1	4.2	4.2									
	2004	3.0	3.6	4.0	4.5	4.7	4.8	4.8	4.8	4.9								
	2005	3.3	3.8	4.2	4.6	5.0	5.2	5.3	5.3	5.3	5.3							
	2006	3.6	4.2	4.6	4.9	5.2	5.7	5.8	5.9	5.9	5.9	6.0						
	2007	4.5	5.0	5.4	5.8	6.1	6.4	6.8	6.9	7.0	7.0	7.0	7.0					
	2008	5.1	5.8	6.2	6.5	6.8	7.1	7.4	7.7	7.8	7.9	7.9	7.9	7.9				
	2009	5.4	6.1	6.8	7.0	7.3	7.6	7.9	8.2	8.5	8.6	8.6	8.6	8.7	8.7			
	2010	6.2	7.0	7.5	8.0	8.3	8.5	8.8	9.0	9.3	9.6	9.7	9.8	9.8	9.8	9.8		

	2011	7.0	7.8	8.4	8.8	9.2	9.5	9.7	10.0	10.2	10.4	10.8	10.8	10.9	10.9	10.9	10.9	
	2012	7.1	8.2	8.9	9.4	9.7	10.1	10.2	10.4	10.7	10.9	11.2	11.5	11.5	11.6	11.6	11.6	11.7
Hypertension	2001	1.9	2.8	3.5	3.8	4.2	4.3											
	2002	2.6	3.2	3.9	4.5	4.8	5.2	5.3										
	2003	3.4	4.3	4.7	5.3	5.8	6.1	6.4	6.6									
	2004	3.5	5.0	5.6	6.0	6.5	7.0	7.2	7.5	7.7								
	2005	4.1	5.3	6.4	7.0	7.3	7.7	8.2	8.4	8.8	8.9							
	2006	4.8	6.4	7.3	8.2	8.7	8.9	9.3	9.7	9.9	10.2	10.3						
	2007	5.9	7.5	8.7	9.2	10.0	10.3	10.5	10.9	11.3	11.5	11.8	11.9					
	2008	6.7	9.1	10.1	11.0	11.5	12.1	12.4	12.5	12.9	13.2	13.4	13.7	13.8				
	2009	6.4	9.3	11.2	12.1	12.8	13.2	13.7	14.0	14.1	14.5	14.7	15.0	15.2	15.3			
	2010	7.4	9.8	11.7	13.2	13.9	14.5	14.9	15.4	15.6	15.8	16.0	16.3	16.5	16.7	16.7		
	2011	8.2	10.9	12.6	14.0	15.3	15.9	16.5	16.8	17.3	17.5	17.6	17.9	18.2	18.3	18.5	18.6	
	2012	7.5	11.2	13.2	14.6	15.8	17.0	17.5	18.0	18.3	18.7	18.9	19.1	19.3	19.5	19.7	19.9	19.9
Kidney Diseases	2001	0.5	0.9	1.3	1.8	2.1	2.3											
	2002	0.7	1.1	1.4	1.8	2.3	2.6	2.7										
	2003	1.0	1.4	1.7	2.0	2.4	2.9	3.2	3.4									
	2004	1.5	2.1	2.5	2.8	3.1	3.5	3.9	4.2	4.4								
	2005	1.5	2.4	3.0	3.3	3.6	3.9	4.3	4.7	5.0	5.2							
	2006	2.0	2.8	3.7	4.2	4.5	4.8	5.1	5.5	5.8	6.2	6.3						
	2007	2.2	3.3	4.1	4.9	5.4	5.6	5.9	6.2	6.5	6.9	7.2	7.4					
	2008	2.7	3.8	4.8	5.5	6.3	6.7	7.0	7.2	7.5	7.9	8.3	8.5	8.7				
	2009	2.6	4.0	4.9	5.9	6.5	7.3	7.7	7.9	8.2	8.4	8.8	9.2	9.4	9.6			
	2010	2.3	3.6	4.9	5.7	6.7	7.3	8.0	8.3	8.6	8.8	9.1	9.5	9.8	10.1	10.2		
	2011	2.2	3.3	4.5	5.6	6.5	7.5	8.0	8.7	9.1	9.4	9.6	9.8	10.2	10.6	10.8	11.0	
	2012	2.3	3.3	4.2	5.2	6.3	7.2	8.1	8.7	9.4	9.7	10.0	10.2	10.5	10.9	11.2	11.5	11.6
Liver Diseases	2001	1.5	2.2	3.0	3.8	4.3	4.7											
	2002	2.1	3.4	4.1	4.9	5.5	6.0	6.4										
	2003	3.0	4.5	5.6	6.3	7.0	7.6	8.1	8.5									
	2004	3.3	5.5	6.7	7.7	8.3	9.0	9.6	10.1	10.4								
	2005	3.9	5.9	7.8	8.9	9.8	10.4	11.0	11.6	12.0	12.3							
	2006	3.9	6.3	8.1	9.7	10.7	11.6	12.2	12.8	13.3	13.7	14.0						
	2007	3.6	6.2	8.3	10.0	11.5	12.5	13.3	13.9	14.5	14.9	15.4	15.6					
	2008	3.9	6.0	8.3	10.2	11.8	13.2	14.1	15.0	15.5	16.0	16.5	16.9	17.2				
	2009	5.1	7.1	8.8	10.8	12.4	13.9	15.3	16.2	16.9	17.4	18.0	18.4	18.8	19.0			
	2010	5.2	8.2	9.9	11.4	13.1	14.6	15.9	17.2	18.1	18.8	19.2	19.8	20.2	20.5	20.8		
	2011	4.6	8.1	10.6	12.0	13.4	15.0	16.4	17.6	18.9	19.7	20.5	20.8	21.4	21.7	22.1	22.3	
	2012	4.1	7.1	10.0	12.2	13.6	14.8	16.3	17.8	18.9	20.2	20.9	21.7	22.0	22.5	22.9	23.3	23.5
Osteoarthritis	2001	0.8	1.3	1.7	2.2	2.5	2.8											
	2002	0.7	1.2	1.7	2.1	2.5	2.8	3.1										
	2003	1.0	1.4	1.8	2.3	2.7	3.1	3.4	3.6									
	2004	1.1	1.7	2.1	2.4	2.8	3.2	3.6	3.9	4.2								
	2005	1.2	1.9	2.4	2.8	3.0	3.4	3.8	4.3	4.5	4.8							
	2006	1.3	2.0	2.5	3.0	3.3	3.6	4.0	4.4	4.8	5.0	5.2						
	2007	1.4	2.3	2.8	3.3	3.8	4.1	4.3	4.7	5.0	5.4	5.7	5.9					
	2008	1.4	2.3	3.0	3.5	4.0	4.5	4.8	5.0	5.3	5.7	6.1	6.3	6.5				
	2009	1.6	2.4	3.2	3.8	4.3	4.8	5.2	5.5	5.7	6.0	6.4	6.8	7.0	7.2			
	2010	1.9	2.7	3.5	4.2	4.9	5.2	5.6	6.1	6.3	6.6	6.9	7.3	7.6	7.9	8.1		
	2011	1.5	2.8	3.5	4.2	5.0	5.5	5.8	6.2	6.7	6.9	7.1	7.5	7.8	8.2	8.4	8.7	
	2012	1.6	2.4	3.7	4.3	5.0	5.7	6.3	6.6	7.0	7.4	7.6	7.8	8.2	8.5	8.9	9.0	9.3

Note: COPD: Chronic Obstructive Pulmonary Diseases.

Supplemental Table 9. Annual trends in prevalence of chronic diseases (as percentage) among HIV-negative individuals in British Columbia from 2001 to 2012 using varying lookback windows, used to construct Supplemental Figure 2.

Chronic Disease	Year	Lookback Window																
		0	1	2	3	4	5	6	7	8	9	10	11	12	13	14	15	16
Alzheimer's/ and/or Dementia	2001	0.1	0.1	0.1	0.1	0.1	0.1											
	2002	0.02	0.1	0.1	0.1	0.1	0.1	0.1										
	2003	0.05	0.05	0.1	0.1	0.1	0.1	0.1	0.1									
	2004	0.1	0.1	0.1	0.1	0.1	0.1	0.2	0.2	0.2								
	2005	0.1	0.1	0.1	0.1	0.1	0.1	0.1	0.2	0.2	0.2							
	2006	0.1	0.1	0.1	0.1	0.1	0.1	0.1	0.1	0.2	0.2	0.2						
	2007	0.1	0.1	0.1	0.1	0.1	0.1	0.2	0.2	0.2	0.2	0.2	0.2					
	2008	0.1	0.1	0.1	0.2	0.2	0.2	0.2	0.2	0.2	0.2	0.3	0.3	0.3				
	2009	0.1	0.2	0.2	0.2	0.2	0.2	0.2	0.2	0.2	0.3	0.3	0.3	0.3	0.3			
	2010	0.1	0.2	0.2	0.2	0.2	0.2	0.2	0.3	0.3	0.3	0.3	0.3	0.3	0.3	0.3		
	2011	0.2	0.2	0.2	0.3	0.3	0.3	0.3	0.3	0.3	0.3	0.3	0.3	0.3	0.4	0.4	0.4	
	2012	0.2	0.2	0.3	0.3	0.3	0.3	0.3	0.3	0.3	0.4	0.4	0.4	0.4	0.4	0.4	0.4	0.4
COPD	2001	0.2	0.3	0.5	0.5	0.6	0.6											
	2002	0.3	0.4	0.5	0.6	0.7	0.7	0.8										
	2003	0.2	0.4	0.5	0.6	0.7	0.8	0.8	0.9									
	2004	0.3	0.4	0.6	0.7	0.8	0.9	0.9	0.9	1.0								
	2005	0.3	0.5	0.6	0.7	0.8	0.9	1.0	1.1	1.1	1.1							
	2006	0.3	0.5	0.6	0.7	0.9	1.0	1.1	1.1	1.2	1.2	1.3						
	2007	0.4	0.6	0.7	0.8	0.9	1.1	1.2	1.2	1.3	1.4	1.4	1.4					
	2008	0.4	0.7	0.8	0.9	1.0	1.1	1.2	1.3	1.4	1.5	1.5	1.5	1.6				
	2009	0.6	0.8	1.0	1.1	1.2	1.3	1.4	1.5	1.6	1.7	1.8	1.8	1.8	1.9			
	2010	0.9	1.2	1.4	1.5	1.6	1.7	1.8	1.9	2.0	2.1	2.1	2.2	2.2	2.2	2.3		
	2011	1.1	1.4	1.6	1.8	1.9	2.0	2.1	2.2	2.2	2.3	2.4	2.5	2.5	2.6	2.6	2.6	
	2012	1.0	1.5	1.8	2.0	2.1	2.3	2.3	2.4	2.5	2.6	2.6	2.7	2.8	2.8	2.9	2.9	2.9
Cardiovascular Diseases	2001	0.5	0.8	0.9	1.1	1.2	1.3											
	2002	0.5	0.8	1.1	1.2	1.4	1.5	1.6										
	2003	0.5	0.9	1.2	1.4	1.5	1.7	1.8	1.9									
	2004	0.7	1.0	1.3	1.6	1.8	1.9	2.0	2.2	2.2								
	2005	0.6	1.1	1.4	1.7	1.9	2.1	2.2	2.4	2.5	2.6							
	2006	0.6	1.1	1.5	1.8	2.0	2.2	2.5	2.6	2.7	2.8	2.9						
	2007	0.7	1.2	1.5	1.9	2.2	2.4	2.6	2.8	3.0	3.1	3.2	3.2					
	2008	0.9	1.4	1.7	2.1	2.4	2.7	2.9	3.1	3.3	3.4	3.5	3.6	3.7				
	2009	0.9	1.5	1.9	2.3	2.6	2.9	3.1	3.4	3.6	3.7	3.8	3.9	4.0	4.1			
	2010	1.0	1.6	2.1	2.5	2.8	3.1	3.4	3.7	3.9	4.0	4.2	4.3	4.4	4.5	4.5		
	2011	1.2	1.8	2.3	2.7	3.1	3.4	3.7	4.0	4.2	4.4	4.6	4.7	4.8	4.9	5.0	5.0	
	2012	1.2	2.0	2.5	3.0	3.4	3.8	4.0	4.3	4.6	4.8	5.0	5.1	5.2	5.3	5.4	5.5	5.6
Diabetes Mellitus	2001	2.0	2.2	2.4	2.5	2.6	2.6											
	2002	2.3	2.6	2.7	2.9	3.0	3.1	3.1										
	2003	2.7	3.1	3.2	3.3	3.5	3.6	3.6	3.6									
	2004	3.1	3.4	3.7	3.8	3.9	4.0	4.1	4.2	4.2								
	2005	3.5	3.9	4.1	4.3	4.4	4.5	4.5	4.6	4.7	4.7							
	2006	4.2	4.5	4.7	4.8	5.0	5.1	5.2	5.2	5.3	5.3	5.4						
	2007	4.7	5.2	5.4	5.5	5.6	5.8	5.9	5.9	6.0	6.0	6.1	6.1					
	2008	5.2	5.8	6.1	6.3	6.4	6.5	6.6	6.7	6.8	6.8	6.9	6.9	6.9				
	2009	5.9	6.4	6.8	7.1	7.2	7.4	7.4	7.6	7.6	7.7	7.7	7.8	7.8	7.8			
	2010	6.5	7.1	7.4	7.7	8.0	8.1	8.2	8.2	8.3	8.4	8.5	8.5	8.6	8.6	8.6		

	2011	7.0	7.7	8.1	8.4	8.7	8.9	9.0	9.1	9.1	9.2	9.3	9.3	9.4	9.4	9.4	9.5	
	2012	7.1	8.0	8.5	8.8	9.1	9.4	9.5	9.6	9.7	9.8	9.9	9.9	10.0	10.0	10.1	10.1	10.1
Hypertension	2001	3.7	4.7	5.4	5.8	6.2	6.5											
	2002	4.3	5.4	6.1	6.6	7.0	7.4	7.6										
	2003	4.8	6.1	7.0	7.5	7.9	8.3	8.6	8.8									
	2004	5.5	7.0	7.9	8.6	9.0	9.4	9.7	10.0	10.1								
	2005	6.2	7.9	9.0	9.7	10.3	10.6	11.0	11.2	11.5	11.6							
	2006	7.3	9.1	10.2	11.0	11.6	12.1	12.4	12.7	12.9	13.1	13.3						
	2007	8.0	9.9	11.2	12.1	12.7	13.3	13.7	14.0	14.2	14.4	14.6	14.8					
	2008	9.0	11.0	12.3	13.2	14.0	14.6	15.0	15.4	15.6	15.9	16.0	16.2	16.3				
	2009	10.0	12.2	13.5	14.4	15.2	15.9	16.4	16.8	17.1	17.4	17.6	17.8	17.9	18.0			
	2010	10.4	12.9	14.4	15.4	16.2	17.0	17.6	18.0	18.4	18.7	18.9	19.1	19.2	19.4	19.5		
	2011	11.1	13.7	15.3	16.5	17.4	18.1	18.7	19.3	19.6	20.0	20.2	20.4	20.6	20.7	20.9	21.0	
	2012	10.9	13.9	15.8	17.1	18.2	18.9	19.6	20.1	20.6	21.0	21.3	21.5	21.7	21.9	22.0	22.1	22.2
Kidney Diseases	2001	0.1	0.2	0.2	0.2	0.3	0.3											
	2002	0.1	0.2	0.2	0.2	0.3	0.3	0.3										
	2003	0.2	0.2	0.2	0.3	0.3	0.3	0.4	0.4									
	2004	0.2	0.2	0.3	0.3	0.4	0.4	0.4	0.4	0.5								
	2005	0.2	0.3	0.3	0.4	0.4	0.4	0.5	0.5	0.5	0.5							
	2006	0.2	0.3	0.4	0.4	0.4	0.5	0.5	0.5	0.6	0.6	0.6						
	2007	0.3	0.3	0.4	0.5	0.5	0.6	0.6	0.6	0.6	0.7	0.7	0.7					
	2008	0.4	0.5	0.5	0.6	0.6	0.7	0.7	0.7	0.8	0.8	0.8	0.8	0.9				
	2009	0.5	0.6	0.6	0.7	0.8	0.8	0.9	0.9	0.9	0.9	1.0	1.0	1.0	1.0			
	2010	0.5	0.6	0.7	0.8	0.9	0.9	1.0	1.0	1.0	1.1	1.1	1.1	1.1	1.2	1.2		
	2011	0.6	0.7	0.9	0.9	1.0	1.1	1.1	1.2	1.2	1.2	1.2	1.3	1.3	1.3	1.4	1.4	
	2012	0.6	0.8	0.9	1.1	1.2	1.2	1.3	1.3	1.4	1.4	1.4	1.4	1.5	1.5	1.5	1.6	1.6
Liver Diseases	2001	0.2	0.3	0.4	0.5	0.5	0.5											
	2002	0.2	0.3	0.4	0.5	0.6	0.6	0.7										
	2003	0.2	0.3	0.4	0.5	0.6	0.7	0.7	0.7									
	2004	0.2	0.3	0.5	0.6	0.7	0.7	0.8	0.9	0.9								
	2005	0.2	0.3	0.5	0.6	0.7	0.8	0.8	0.9	1.0	1.0							
	2006	0.2	0.3	0.5	0.6	0.7	0.8	0.9	1.0	1.0	1.1	1.1						
	2007	0.3	0.4	0.5	0.6	0.7	0.8	0.9	1.0	1.1	1.2	1.2	1.2					
	2008	0.2	0.4	0.5	0.6	0.8	0.9	0.9	1.0	1.1	1.2	1.3	1.3	1.3				
	2009	0.3	0.5	0.6	0.7	0.8	1.0	1.0	1.1	1.2	1.3	1.4	1.4	1.5	1.5			
	2010	0.4	0.6	0.7	0.8	1.0	1.0	1.1	1.2	1.3	1.4	1.5	1.5	1.6	1.7	1.7		
	2011	0.4	0.6	0.8	0.9	1.0	1.1	1.2	1.3	1.4	1.5	1.6	1.7	1.7	1.8	1.8	1.8	
	2012	0.5	0.7	0.9	1.1	1.2	1.3	1.4	1.5	1.6	1.7	1.7	1.8	1.9	2.0	2.0	2.1	2.1
Osteoarthritis	2001	0.6	1.0	1.4	1.7	2.0	2.2											
	2002	0.7	1.2	1.5	1.8	2.1	2.4	2.6										
	2003	0.8	1.3	1.7	2.0	2.3	2.6	2.9	3.0									
	2004	1.0	1.5	1.9	2.2	2.5	2.9	3.1	3.4	3.6								
	2005	1.0	1.7	2.1	2.5	2.8	3.1	3.4	3.6	3.9	4.1							
	2006	1.2	1.8	2.4	2.7	3.1	3.4	3.7	4.0	4.2	4.5	4.6						
	2007	1.3	2.0	2.6	3.1	3.4	3.8	4.1	4.3	4.6	4.8	5.0	5.2					
	2008	1.4	2.2	2.8	3.3	3.8	4.1	4.4	4.7	4.9	5.2	5.4	5.6	5.8				
	2009	1.5	2.4	3.1	3.6	4.1	4.5	4.8	5.2	5.4	5.6	5.9	6.1	6.3	6.4			
	2010	1.6	2.5	3.3	3.9	4.4	4.9	5.3	5.5	5.9	6.1	6.3	6.5	6.7	6.9	7.1		
	2011	1.8	2.7	3.6	4.3	4.8	5.3	5.7	6.1	6.3	6.6	6.8	7.1	7.3	7.4	7.6	7.8	
	2012	1.8	2.9	3.7	4.5	5.1	5.6	6.1	6.5	6.8	7.1	7.3	7.6	7.8	8.0	8.1	8.3	8.5

Note: COPD: Chronic Obstructive Pulmonary Diseases.

Supplemental Table 10. Annual trends in incidence of chronic diseases (as percentage) among people living with HIV in British Columbia from 2001 to 2012 using varying lookback windows, used to construct Figure 5.

Chronic Disease	Year	Lookback Window																
		0	1	2	3	4	5	6	7	8	9	10	11	12	13	14	15	16
Alzheimer's/ and/or Dementia	2001	0.2	0.2	0.1	0.1	0.1	0.1											
	2002	0.4	0.2	0.2	0.2	0.2	0.2	0.2										
	2003	0.3	0.2	0.2	0.2	0.2	0.2	0.2	0.2									
	2004	0.4	0.2	0.2	0.2	0.2	0.2	0.2	0.2	0.2								
	2005	0.3	0.1	0.1	0.1	0.1	0.1	0.1	0.1	0.2	0.2							
	2006	0.6	0.5	0.5	0.5	0.5	0.5	0.5	0.5	0.5	0.5	0.5						
	2007	0.3	0.1	0.1	0.1	0.1	0.1	0.1	0.1	0.1	0.1	0.1	0.1					
	2008	0.5	0.4	0.3	0.3	0.3	0.3	0.3	0.3	0.3	0.3	0.3	0.3	0.3				
	2009	0.7	0.5	0.4	0.4	0.4	0.4	0.4	0.4	0.4	0.4	0.4	0.4	0.4	0.4			
	2010	0.6	0.2	0.2	0.2	0.2	0.2	0.2	0.2	0.2	0.2	0.2	0.2	0.2	0.2	0.2		
	2011	0.6	0.3	0.3	0.2	0.2	0.2	0.2	0.2	0.2	0.2	0.2	0.2	0.2	0.2	0.2	0.2	
	2012	0.7	0.4	0.3	0.3	0.3	0.3	0.3	0.3	0.3	0.3	0.3	0.3	0.3	0.3	0.3	0.3	0.3
COPD	2001	0.1	0.1	0.1	0.1	0.1	0.1											
	2002	0.3	0.2	0.2	0.2	0.2	0.2	0.2										
	2003	0.5	0.5	0.4	0.4	0.4	0.4	0.4	0.4									
	2004	0.7	0.6	0.5	0.5	0.5	0.5	0.5	0.5	0.5								
	2005	0.6	0.4	0.4	0.4	0.4	0.3	0.3	0.3	0.3	0.3							
	2006	0.8	0.6	0.5	0.5	0.5	0.5	0.5	0.5	0.5	0.5	0.5						
	2007	1.0	0.7	0.6	0.6	0.6	0.6	0.6	0.5	0.5	0.5	0.5	0.5					
	2008	1.4	0.9	0.9	0.8	0.8	0.8	0.8	0.8	0.8	0.7	0.7	0.7	0.7				
	2009	1.9	1.5	1.4	1.4	1.3	1.3	1.3	1.3	1.3	1.3	1.3	1.3	1.3	1.3			
	2010	2.6	1.6	1.4	1.3	1.3	1.3	1.3	1.3	1.3	1.3	1.3	1.2	1.2	1.2	1.2		
	2011	2.7	1.6	1.4	1.2	1.2	1.1	1.1	1.1	1.1	1.1	1.1	1.1	1.1	1.1	1.1	1.1	
	2012	3.2	1.6	1.2	1.1	1.1	1.0	1.0	1.0	1.0	1.0	1.0	1.0	1.0	1.0	1.0	1.0	1.0
Cardiovascular Diseases	2001	0.6	0.4	0.4	0.3	0.3	0.3											
	2002	0.7	0.5	0.5	0.5	0.4	0.4	0.4										
	2003	0.6	0.4	0.4	0.3	0.3	0.3	0.3	0.3									
	2004	0.9	0.7	0.6	0.6	0.6	0.5	0.5	0.5	0.5								
	2005	0.7	0.6	0.5	0.5	0.5	0.5	0.5	0.4	0.4	0.4							
	2006	1.1	0.8	0.7	0.7	0.7	0.7	0.7	0.7	0.7	0.7	0.7						
	2007	1.2	0.9	0.8	0.8	0.7	0.7	0.6	0.6	0.6	0.6	0.6	0.6					
	2008	1.2	0.9	0.8	0.7	0.7	0.7	0.6	0.6	0.6	0.6	0.6	0.6	0.6				
	2009	1.3	0.9	0.9	0.8	0.7	0.7	0.7	0.7	0.7	0.7	0.7	0.7	0.7	0.7			
	2010	1.4	0.9	0.9	0.8	0.8	0.7	0.7	0.7	0.7	0.7	0.6	0.6	0.6	0.6	0.6		
	2011	1.7	1.1	1.0	1.0	1.0	0.9	0.9	0.9	0.8	0.8	0.8	0.8	0.8	0.8	0.8	0.8	
	2012	1.7	1.2	1.1	1.1	1.0	1.0	1.0	0.9	0.9	0.9	0.9	0.9	0.9	0.9	0.9	0.9	0.9
Diabetes Mellitus	2001	2.5	1.0	1.0	1.0	1.0	1.0											
	2002	2.6	0.8	0.7	0.7	0.7	0.7	0.7										
	2003	2.9	0.7	0.6	0.6	0.6	0.6	0.6	0.6									
	2004	3.0	0.8	0.7	0.7	0.7	0.7	0.7	0.7	0.7								
	2005	3.3	0.8	0.6	0.6	0.5	0.5	0.5	0.5	0.5	0.5							
	2006	3.6	0.9	0.8	0.8	0.7	0.7	0.7	0.7	0.7	0.7	0.7						
	2007	4.5	1.5	1.3	1.2	1.2	1.2	1.2	1.2	1.1	1.1	1.1	1.1					
	2008	5.1	1.4	1.2	1.1	1.1	1.1	1.0	1.0	1.0	1.0	1.0	1.0	1.0				
	2009	5.4	1.0	1.0	0.9	0.9	0.9	0.9	0.9	0.8	0.8	0.8	0.8	0.8	0.8			
	2010	6.2	1.7	1.5	1.3	1.3	1.3	1.3	1.3	1.2	1.2	1.2	1.2	1.2	1.2	1.2		

	2011	7.0	1.7	1.4	1.3	1.3	1.3	1.3	1.3	1.3	1.3	1.3	1.2	1.2	1.2	1.2	1.2	
	2012	7.1	1.4	1.2	1.1	1.0	0.9	0.8	0.8	0.8	0.8	0.8	0.8	0.8	0.8	0.8	0.8	0.8
Hypertension	2001	1.9	0.7	0.6	0.6	0.6	0.6											
	2002	2.6	1.4	1.2	1.1	1.0	1.0	1.0										
	2003	3.4	1.8	1.6	1.5	1.4	1.4	1.4	1.4									
	2004	3.5	1.6	1.4	1.3	1.3	1.2	1.2	1.2	1.2								
	2005	4.1	1.9	1.6	1.5	1.4	1.3	1.4	1.4	1.4	1.4							
	2006	4.8	2.4	2.1	1.9	1.8	1.8	1.7	1.7	1.7	1.6	1.6						
	2007	5.9	2.9	2.4	2.1	2.0	1.8	1.8	1.8	1.8	1.8	1.8	1.8					
	2008	6.7	3.4	2.8	2.5	2.5	2.3	2.3	2.3	2.2	2.2	2.2	2.1	2.1				
	2009	6.4	2.8	2.4	2.2	2.1	2.0	1.9	1.9	1.8	1.8	1.8	1.8	1.8	1.7			
	2010	7.4	3.6	2.6	2.3	2.1	2.0	2.0	2.0	1.9	1.9	1.9	1.8	1.8	1.8	1.7		
	2011	8.2	3.8	3.1	2.7	2.4	2.3	2.3	2.3	2.3	2.3	2.3	2.3	2.3	2.3	2.3	2.3	
	2012	7.5	3.3	2.6	2.2	2.1	2.0	1.9	1.9	1.8	1.8	1.7	1.7	1.7	1.7	1.7	1.7	1.7
Kidney Diseases	2001	0.5	0.3	0.3	0.3	0.3	0.3											
	2002	0.7	0.5	0.5	0.5	0.5	0.5	0.5										
	2003	1.0	0.7	0.6	0.6	0.6	0.6	0.6	0.6									
	2004	1.5	1.2	1.1	1.1	1.1	1.1	1.1	1.0	1.0								
	2005	1.5	0.9	0.9	0.9	0.8	0.8	0.8	0.8	0.8	0.8							
	2006	2.0	1.4	1.3	1.2	1.2	1.2	1.2	1.2	1.2	1.2	1.2						
	2007	2.2	1.3	1.3	1.2	1.2	1.2	1.2	1.1	1.1	1.2	1.1	1.1					
	2008	2.7	1.6	1.5	1.5	1.5	1.4	1.4	1.4	1.4	1.4	1.4	1.4	1.4				
	2009	2.6	1.4	1.2	1.2	1.1	1.1	1.0	1.0	1.0	1.0	1.0	1.0	1.0	1.0			
	2010	2.3	1.0	0.9	0.9	0.8	0.8	0.7	0.7	0.7	0.7	0.7	0.7	0.7	0.8	0.8		
	2011	2.2	1.0	0.9	0.8	0.8	0.8	0.8	0.8	0.8	0.8	0.8	0.8	0.8	0.8	0.8	0.8	
	2012	2.3	1.1	0.9	0.8	0.7	0.7	0.7	0.7	0.7	0.7	0.7	0.7	0.7	0.7	0.7	0.7	0.7
Liver Diseases	2001	1.5	1.3	1.3	1.2	1.2	1.1											
	2002	2.1	1.9	1.9	1.9	1.8	1.7	1.7										
	2003	3.0	2.5	2.3	2.3	2.3	2.2	2.2	2.2									
	2004	3.3	2.6	2.3	2.2	2.2	2.1	2.1	2.1	2.1								
	2005	3.9	2.7	2.4	2.3	2.3	2.2	2.2	2.2	2.2	2.2							
	2006	3.9	2.5	2.3	2.1	2.0	2.0	2.0	2.0	1.9	1.9	1.9						
	2007	3.6	2.3	2.2	2.1	2.0	2.0	2.0	1.9	1.9	1.9	1.9	1.9					
	2008	3.9	2.5	2.2	2.1	2.0	1.9	1.9	1.9	1.8	1.8	1.8	1.8	1.8				
	2009	5.1	3.4	3.0	2.8	2.5	2.4	2.4	2.4	2.3	2.3	2.3	2.3	2.2	2.2			
	2010	5.2	3.3	2.9	2.8	2.6	2.5	2.3	2.3	2.3	2.2	2.2	2.2	2.2	2.2	2.2		
	2011	4.6	3.1	2.6	2.4	2.3	2.1	2.1	2.1	2.0	2.1	2.0	2.0	2.0	2.0	1.9	1.9	
	2012	4.1	2.6	2.1	1.8	1.7	1.7	1.6	1.6	1.6	1.6	1.5	1.5	1.5	1.5	1.5	1.5	1.5
Osteoarthritis	2001	0.8	0.5	0.5	0.4	0.4	0.4											
	2002	0.7	0.4	0.4	0.3	0.3	0.3	0.3										
	2003	1.0	0.7	0.6	0.6	0.6	0.6	0.6	0.6									
	2004	1.1	0.7	0.6	0.6	0.6	0.6	0.6	0.5	0.5								
	2005	1.2	0.8	0.8	0.7	0.7	0.7	0.6	0.6	0.6	0.6							
	2006	1.3	0.8	0.7	0.6	0.6	0.6	0.5	0.5	0.5	0.5	0.5						
	2007	1.4	1.0	0.8	0.8	0.8	0.8	0.8	0.7	0.7	0.7	0.7	0.7					
	2008	1.4	0.9	0.8	0.8	0.7	0.7	0.7	0.7	0.7	0.7	0.7	0.7	0.7				
	2009	1.6	1.0	0.9	0.8	0.8	0.8	0.8	0.8	0.8	0.8	0.8	0.7	0.7	0.7			
	2010	1.9	1.2	1.1	1.1	1.0	0.9	0.9	0.9	0.9	0.9	0.9	0.9	0.9	0.9	0.9		
	2011	1.5	0.9	0.8	0.8	0.8	0.7	0.7	0.6	0.6	0.6	0.6	0.6	0.6	0.6	0.6	0.6	
	2012	1.6	0.9	0.9	0.9	0.8	0.8	0.8	0.8	0.8	0.7	0.8	0.8	0.8	0.7	0.7	0.7	0.7

Note: COPD: Chronic Obstructive Pulmonary Diseases.

Supplemental Table 11. Annual trends in incidence of chronic diseases (as percentage) among HIV-negative individuals in British Columbia from 2001 to 2012 using varying lookback windows, used to construct Supplemental Figure 3.

Chronic Disease	Year	Lookback Window																
		0	1	2	3	4	5	6	7	8	9	10	11	12	13	14	15	16
Alzheimer's/ and/or Dementia	2001	0.1	0.05	0.05	0.05	0.05	0.05											
	2002	0.03	0.01	0.01	0.01	0.01	0.01	0.01										
	2003	0.05	0.02	0.02	0.01	0.01	0.01	0.01	0.01									
	2004	0.1	0.03	0.03	0.03	0.03	0.03	0.03	0.03	0.03								
	2005	0.1	0.02	0.02	0.02	0.02	0.02	0.02	0.02	0.02	0.02							
	2006	0.1	0.02	0.01	0.01	0.01	0.01	0.01	0.01	0.01	0.01	0.01						
	2007	0.1	0.03	0.03	0.03	0.03	0.03	0.03	0.03	0.03	0.03	0.03	0.03					
	2008	0.1	0.1	0.1	0.1	0.1	0.1	0.1	0.1	0.1	0.1	0.1	0.1	0.1				
	2009	0.1	0.03	0.03	0.03	0.03	0.03	0.03	0.03	0.03	0.03	0.03	0.03	0.03	0.03			
	2010	0.1	0.05	0.05	0.05	0.05	0.05	0.05	0.05	0.05	0.05	0.05	0.05	0.05	0.05	0.05		
	2011	0.2	0.1	0.1	0.1	0.1	0.1	0.1	0.1	0.1	0.1	0.1	0.1	0.1	0.1	0.1	0.1	
	2012	0.2	0.1	0.1	0.1	0.1	0.1	0.1	0.1	0.1	0.1	0.1	0.1	0.1	0.1	0.1	0.1	0.1
COPD	2001	0.2	0.2	0.2	0.2	0.2	0.2											
	2002	0.3	0.2	0.2	0.2	0.2	0.2	0.2										
	2003	0.2	0.2	0.1	0.1	0.1	0.1	0.1	0.1									
	2004	0.3	0.2	0.2	0.2	0.2	0.2	0.2	0.1	0.1								
	2005	0.3	0.2	0.2	0.2	0.2	0.2	0.2	0.2	0.2	0.2							
	2006	0.3	0.2	0.2	0.2	0.2	0.2	0.2	0.2	0.2	0.2	0.2						
	2007	0.4	0.3	0.2	0.2	0.2	0.2	0.2	0.2	0.2	0.2	0.2	0.2					
	2008	0.4	0.3	0.2	0.2	0.2	0.2	0.2	0.2	0.2	0.2	0.2	0.2	0.2				
	2009	0.6	0.4	0.4	0.4	0.3	0.3	0.3	0.3	0.3	0.3	0.3	0.3	0.3	0.3			
	2010	0.9	0.6	0.5	0.5	0.5	0.5	0.5	0.5	0.5	0.5	0.5	0.5	0.5	0.5	0.5		
	2011	1.1	0.5	0.5	0.4	0.4	0.4	0.4	0.4	0.4	0.4	0.4	0.4	0.4	0.4	0.4	0.4	
	2012	1.0	0.4	0.4	0.4	0.4	0.4	0.4	0.4	0.4	0.4	0.3	0.3	0.3	0.3	0.3	0.3	0.3
Cardiovascular Diseases	2001	0.5	0.3	0.3	0.3	0.3	0.3											
	2002	0.5	0.3	0.3	0.3	0.3	0.3	0.3										
	2003	0.5	0.4	0.4	0.4	0.3	0.3	0.3	0.3									
	2004	0.7	0.5	0.4	0.4	0.4	0.4	0.4	0.4	0.4								
	2005	0.6	0.4	0.4	0.3	0.3	0.3	0.3	0.3	0.3	0.3							
	2006	0.6	0.5	0.4	0.4	0.4	0.3	0.3	0.3	0.3	0.3	0.3						
	2007	0.7	0.6	0.5	0.5	0.4	0.4	0.4	0.4	0.4	0.4	0.4	0.4					
	2008	0.9	0.6	0.6	0.6	0.5	0.5	0.5	0.5	0.5	0.4	0.4	0.4	0.4				
	2009	0.9	0.6	0.6	0.5	0.5	0.5	0.5	0.5	0.5	0.4	0.4	0.4	0.4	0.4			
	2010	1.0	0.7	0.6	0.6	0.6	0.6	0.5	0.5	0.5	0.5	0.5	0.5	0.5	0.5	0.5		
	2011	1.2	0.8	0.7	0.7	0.6	0.6	0.6	0.6	0.6	0.6	0.5	0.5	0.5	0.5	0.5	0.5	
	2012	1.2	0.8	0.8	0.7	0.7	0.7	0.6	0.6	0.6	0.6	0.6	0.6	0.6	0.6	0.6	0.6	0.6
Diabetes Mellitus	2001	2.0	0.6	0.5	0.5	0.5	0.5											
	2002	2.3	0.6	0.6	0.5	0.5	0.5	0.5										
	2003	2.7	0.7	0.6	0.6	0.6	0.6	0.6	0.6									
	2004	3.1	0.8	0.7	0.6	0.6	0.6	0.6	0.6	0.6								
	2005	3.5	0.7	0.7	0.6	0.6	0.5	0.5	0.5	0.5	0.5							
	2006	4.2	1.0	0.8	0.8	0.8	0.8	0.7	0.7	0.7	0.7	0.7						
	2007	4.7	1.1	1.0	0.9	0.9	0.8	0.8	0.8	0.8	0.8	0.8	0.8					
	2008	5.2	1.1	1.0	1.0	0.9	0.9	0.9	0.9	0.9	0.9	0.9	0.9	0.9				
	2009	5.9	1.3	1.1	1.1	1.0	1.0	1.0	1.0	1.0	1.0	1.0	1.0	1.0	1.0			
	2010	6.5	1.3	1.1	1.0	0.9	0.9	0.9	0.9	0.9	0.8	0.8	0.8	0.8	0.8	0.8		

	2011	7.0	1.3	1.1	1.0	1.0	1.0	1.0	1.0	1.0	1.0	0.9	1.0	1.0	0.9	0.9	0.9	
	2012	7.1	1.0	0.9	0.8	0.8	0.8	0.7	0.7	0.7	0.7	0.7	0.7	0.7	0.7	0.7	0.7	0.7
Hypertension	2001	3.7	1.6	1.4	1.3	1.2	1.2											
	2002	4.3	1.8	1.5	1.4	1.3	1.3	1.3										
	2003	4.8	2.0	1.7	1.5	1.4	1.4	1.4	1.4									
	2004	5.5	2.3	1.9	1.8	1.7	1.6	1.6	1.5	1.5								
	2005	6.2	2.6	2.2	2.0	1.9	1.8	1.8	1.8	1.7	1.7							
	2006	7.3	3.1	2.5	2.3	2.1	2.1	2.0	2.0	1.9	1.9	1.9						
	2007	8.0	2.9	2.3	2.1	2.0	1.9	1.8	1.8	1.8	1.8	1.7	1.7					
	2008	9.0	3.2	2.6	2.4	2.2	2.1	2.0	2.0	2.0	1.9	1.9	1.9	1.9				
	2009	10.0	3.5	2.8	2.5	2.3	2.3	2.2	2.2	2.1	2.1	2.1	2.1	2.0	2.0			
	2010	10.4	3.2	2.6	2.2	2.1	2.1	2.0	1.9	1.9	1.9	1.9	1.8	1.8	1.8	1.8		
	2011	11.1	3.6	2.8	2.4	2.3	2.2	2.1	2.0	2.0	2.0	1.9	1.9	1.9	1.9	1.9	1.9	
	2012	10.9	3.2	2.5	2.2	2.0	1.9	1.8	1.8	1.7	1.7	1.7	1.7	1.6	1.6	1.6	1.6	1.6
Kidney Diseases	2001	0.1																
	2002	0.1	0.1															
	2003	0.2	0.1	0.1	0.1	0.1	0.1	0.1	0.1									
	2004	0.2	0.1	0.1	0.1	0.1	0.1	0.1	0.1	0.1								
	2005	0.2	0.1	0.1	0.1	0.1	0.1	0.1	0.1	0.1	0.1							
	2006	0.2	0.1	0.1	0.1	0.1	0.1	0.1	0.1	0.1	0.1	0.1						
	2007	0.3	0.1	0.1	0.1	0.1	0.1	0.1	0.1	0.1	0.1	0.1	0.1					
	2008	0.4	0.2	0.2	0.2	0.2	0.1	0.1	0.1	0.1	0.1	0.1	0.1	0.1				
	2009	0.5	0.2	0.2	0.2	0.2	0.2	0.2	0.2	0.2	0.2	0.2	0.2	0.2	0.2			
	2010	0.5	0.2	0.2	0.2	0.2	0.2	0.2	0.2	0.2	0.2	0.2	0.1	0.1	0.1	0.1		
	2011	0.6	0.2	0.2	0.2	0.2	0.2	0.2	0.2	0.2	0.2	0.2	0.2	0.2	0.2	0.2	0.2	
	2012	0.6	0.2	0.2	0.2	0.2	0.2	0.2	0.2	0.2	0.2	0.2	0.2	0.2	0.2	0.2	0.2	0.2
Liver Diseases	2001	0.2	0.2	0.1	0.1	0.1	0.1											
	2002	0.2	0.1	0.1	0.1	0.1	0.1	0.1										
	2003	0.2	0.1	0.1	0.1	0.1	0.1	0.1	0.1									
	2004	0.2	0.2	0.1	0.1	0.1	0.1	0.1	0.1	0.1								
	2005	0.2	0.1	0.1	0.1	0.1	0.1	0.1	0.1	0.1	0.1							
	2006	0.2	0.2	0.2	0.1	0.1	0.1	0.1	0.1	0.1	0.1	0.1						
	2007	0.3	0.2	0.2	0.1	0.1	0.1	0.1	0.1	0.1	0.1	0.1	0.1					
	2008	0.2	0.2	0.1	0.1	0.1	0.1	0.1	0.1	0.1	0.1	0.1	0.1	0.1				
	2009	0.3	0.2	0.2	0.2	0.2	0.2	0.2	0.2	0.2	0.2	0.2	0.2	0.2	0.2			
	2010	0.4	0.2	0.2	0.2	0.2	0.2	0.2	0.2	0.2	0.2	0.2	0.2	0.2	0.2	0.2		
	2011	0.4	0.3	0.2	0.2	0.2	0.2	0.2	0.2	0.2	0.2	0.2	0.2	0.2	0.2	0.2	0.2	
	2012	0.5	0.3	0.3	0.3	0.3	0.2	0.2	0.2	0.2	0.2	0.2	0.2	0.2	0.2	0.2	0.2	0.2
Osteoarthritis	2001	0.6	0.4	0.4	0.4	0.4	0.4											
	2002	0.7	0.5	0.5	0.4	0.4	0.4	0.4										
	2003	0.8	0.6	0.5	0.5	0.5	0.5	0.4	0.4									
	2004	1.0	0.7	0.6	0.6	0.6	0.6	0.5	0.5	0.5								
	2005	1.0	0.7	0.6	0.6	0.6	0.6	0.6	0.6	0.6	0.6							
	2006	1.2	0.8	0.7	0.7	0.6	0.6	0.6	0.6	0.6	0.6	0.6						
	2007	1.3	0.9	0.8	0.7	0.7	0.7	0.7	0.7	0.6	0.6	0.6	0.6					
	2008	1.4	0.9	0.8	0.7	0.7	0.7	0.7	0.7	0.6	0.6	0.6	0.6	0.6				
	2009	1.5	1.0	0.9	0.8	0.8	0.8	0.8	0.8	0.7	0.7	0.7	0.7	0.7	0.7			
	2010	1.6	1.0	0.9	0.9	0.8	0.8	0.8	0.8	0.7	0.7	0.7	0.7	0.7	0.7	0.7		
	2011	1.8	1.2	1.1	1.0	0.9	0.9	0.9	0.9	0.8	0.8	0.8	0.8	0.8	0.8	0.8	0.8	
	2012	1.8	1.1	1.0	0.9	0.9	0.8	0.8	0.8	0.8	0.8	0.8	0.8	0.8	0.8	0.8	0.8	0.7

Note: COPD: Chronic Obstructive Pulmonary Diseases.

Supplemental Figure 1. Flowchart outlining the derivation of the final 1:5 matched analytical sample of 5,151 PLWH and 25,755 HIV-negative individuals in British Columba, Canada, who had administrative health records or public insurance registration since 1996 and were still alive in 2012.

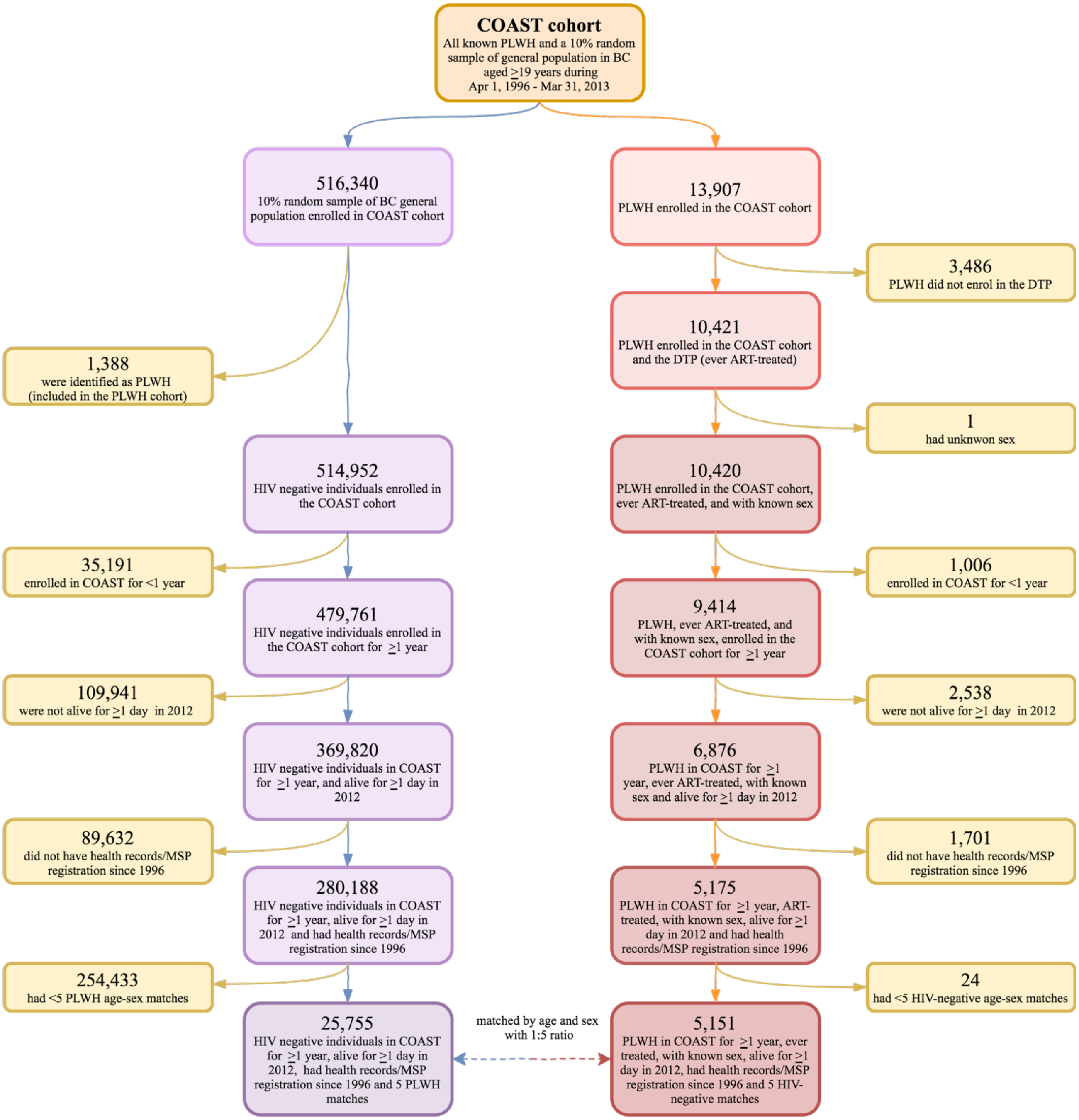

Note: PLWH: people living with HIV; COAST: Comparative Outcomes and Service Utilization Trends; DTP: BC Centre for Excellence in HIV-AIDS Drug Treatment Program; ART: antiretroviral therapy.

Supplemental Figure 2. Annual trends in prevalence of chronic diseases among HIV-negative individuals in British Columbia from 2001 to 2012 using varying lookback windows.

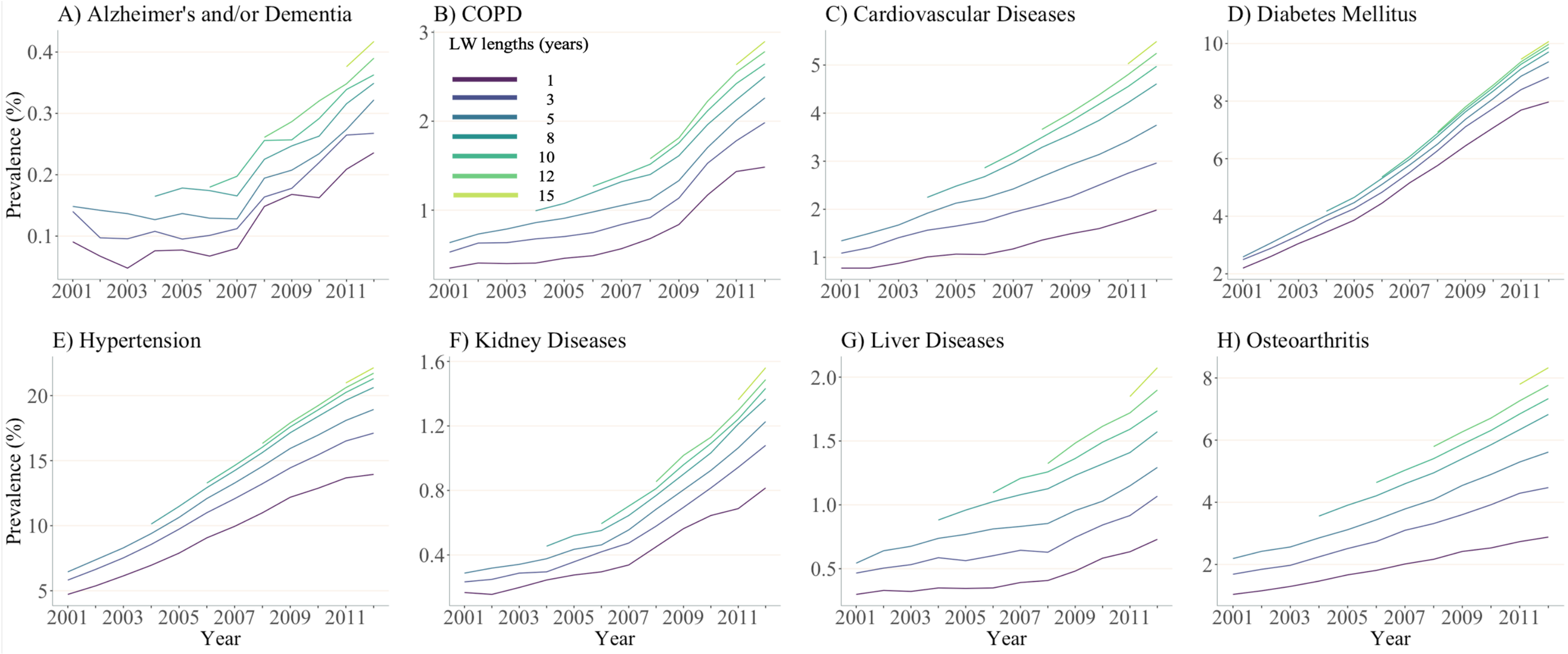

Note: PLWH: people living with HIV; LW: lookback window; COPD: Chronic Obstructive Pulmonary Diseases. Vertical scales differ for each graph for illustration purposes.

Supplemental Figure 3. Annual trends in incidence of chronic diseases among HIV-negative individuals in British Columbia from 2001 to 2012 using varying lookback windows.

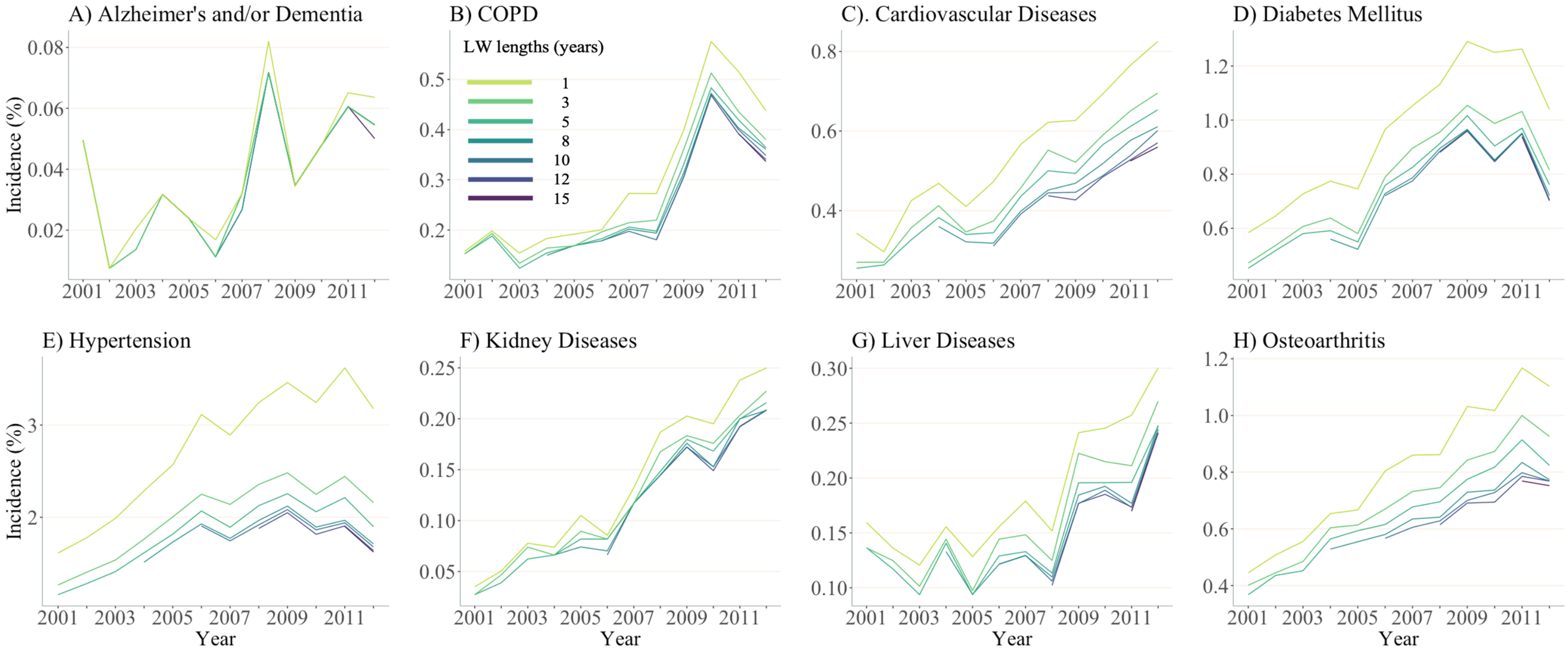

Note: PLWH: people living with HIV; LW: lookback window; COPD: Chronic Obstructive Pulmonary Diseases. Vertical scales differ for each graph for illustration purposes.
